# Robotic *versus* laparoscopic minimally invasive inguinal hernia repair: randomized clinical trial (the ROGER trial)

**DOI:** 10.1093/bjs/znaf283

**Published:** 2026-01-09

**Authors:** Fiorenzo V Angehrn, Julian Süsstrunk, Romano Schneider, Kaspar Baltzer, Beat P Müller, Johannes Baur, Daniel C Steinemann

**Affiliations:** Clarunis, Department of Visceral Surgery, University Digestive Health Care Centre, St Clara Hospital and University Hospital Basel, Basel, Switzerland; Clarunis, Department of Visceral Surgery, University Digestive Health Care Centre, St Clara Hospital and University Hospital Basel, Basel, Switzerland; Clarunis, Department of Visceral Surgery, University Digestive Health Care Centre, St Clara Hospital and University Hospital Basel, Basel, Switzerland; Clarunis, Department of Visceral Surgery, University Digestive Health Care Centre, St Clara Hospital and University Hospital Basel, Basel, Switzerland; Clarunis, Department of Visceral Surgery, University Digestive Health Care Centre, St Clara Hospital and University Hospital Basel, Basel, Switzerland; Clarunis, Department of Visceral Surgery, University Digestive Health Care Centre, St Clara Hospital and University Hospital Basel, Basel, Switzerland; Clarunis, Department of Visceral Surgery, University Digestive Health Care Centre, St Clara Hospital and University Hospital Basel, Basel, Switzerland

## Abstract

**Background:**

Superiority of robotic inguinal hernia repair compared with a laparoscopic minimally invasive approach remains unproven. The aim of this study was to evaluate postoperative pain after laparoscopic totally extraperitoneal repair (TEP) compared with robotic transabdominal preperitoneal repair (rTAPP).

**Methods:**

This was a prospective, patient- and investigator-blinded, two-group, single-centre RCT conducted at a tertiary Swiss healthcare institution including 182 patients undergoing elective primary inguinal hernia repair. Patients were randomized 1 : 1 and stratified according to BMI and unilateral or bilateral hernia to either TEP or rTAPP. Surgery took place between March 2022 and November 2024. The primary endpoint was postoperative pain while coughing 24 h after surgery. Surgical workload (assessed using the National Aeronautics and Space Administration (NASA) Task Load Index (TLX)) was also recorded.

**Results:**

In total, 91 patients (93% male, mean(s.d.) age of 56.8(15.2) years, mean(s.d.) BMI of 24.8(3) kg/m^2^, and 22% with bilateral hernias) were randomized to TEP and 91 patients (95% male, mean(s.d.) age of 55.1(14.5) years, mean(s.d.) BMI of 24.6(2.9) kg/m^2^, and 21% with bilateral hernias) were randomized to rTAPP. Primary outcome data were available for 90 TEP patients and 88 rTAPP patients. The median postoperative pain while coughing on a numeric rating scale 24 h after surgery was 5 (interquartile range (i.q.r.) 2–7) after TEP and 4 (i.q.r. 2–7) after rTAPP (*P* = 0.431, Cohen’s d = 0.12). The mean(s.d.) operating time for unilateral hernias was 64.2(19.2) min for TEP and 80.3(20.9) min for rTAPP (*P* < 0.001). Ten (11%) postoperative complications occured after TEP and nine (10%) after rTAPP (*P* > 0.999). The mean(s.d.) NASA raw TLX score was 34.0(17.2) after TEP and 18.4(10.7) after rTAPP (*P* < 0.001).

**Conclusion:**

rTAPP demonstrated no superiority over TEP regarding postoperative pain and complication rates. rTAPP was associated with a reduced surgeon workload at the expense of a longer operating time.

**Registration number:**

NCT05216276 (http://www.clinicaltrials.gov).

## Teaser

This RCT compared robotic transabdominal preperitoneal repair (rTAPP) with conventional totally extraperitoneal repair (TEP) for inguinal hernia repair. The study found that rTAPP demonstrated no superiority over TEP regarding postoperative pain and complication rates. rTAPP was associated with a reduced surgeon workload at the expense of a longer operating time.

## Introduction

Inguinal hernias are common, with an annual incidence of up to 34/10 000, and their repair is one of the most frequently performed surgical procedures^1^. Surgical management is the only definitive treatment for inguinal hernias and current evidence suggests that minimally invasive repair leads to less postoperative and chronic pain, fewer haematomas and wound infections, and a faster return to work compared with the best available open Lichtenstein technique^2^. Recently released international guidelines endorsed by most hernia societies thus recommend minimally invasive repair using totally extraperitoneal repair (TEP) or transabdominal preperitoneal repair (TAPP) if appropriate surgical expertise is available^3^.

In minimally invasive inguinal hernia surgery, however, the role of robotically assisted procedures is not yet clear. The multicentre, single-blinded RIVAL trial published in 2020 comparing laparoscopic TAPP with robotic TAPP (rTAPP) did not find any clinical benefit to the robotic approach, but a longer operating time and higher costs^4^. This trial, however, was designed as a pilot study and might have been underpowered to draw valid conclusions regarding specific questions such as postoperative pain. The second RCT on this topic, a single-centre comparison between laparoscopic TAPP and rTAPP (ROLAIS) including 139 patients that was published in 2025, showed that rTAPP was associated with reduced surgical stress, fewer complications, a shorter operating time, and reduced inpatient stay^5^.

Recent retrospective analyses comparing laparoscopic and robotic surgery continue to show conflicting results. A systematic review published in 2022 by Solaini *et al*.^6^ and a matched multicentre study from 2024^7^ found that laparoscopic and robotic inguinal hernia repair lead to similar intraoperative and postoperative outcomes, but at a higher cost and with a longer operating time for robotic approaches. Conversely, robotic inguinal hernia repair was associated with fewer complications and a shorter hospital stay in obese patients in a recently published retrospective analysis including 647 patients^8^.

While rTAPP is the more prevalent approach in current robotic practice, the authors chose to compare it with conventional laparoscopic TEP, which has been the established standard procedure at the authors’ institution for several years. This comparison was undertaken to provide a clinically relevant insight based on the authors’ institutional experience. Therefore, the primary objective of this RCT was to determine whether rTAPP results in significantly reduced postoperative pain compared with conventional laparoscopic TEP.

## Methods

The study was conducted in accordance with the principles of the Helsinki declaration and its later amendments, approved by the local ethical committee, and registered at ClinicalTrials.gov (NCT05216276) before the start of the study^9^. All patients provided written informed consent, including informed consent for their data to be recorded, analysed, and published. The study protocol was published in advance^10^.

### Trial design and participants

This study was a prospective, blinded, two-group, single-centre RCT conducted at the Clarunis University Digestive Health Care Centre in Basel, Switzerland, including 182 patients with unilateral or bilateral inguinal hernias. Surgical procedures for this trial were performed between March 2022 and November 2024, with the last date of follow-up for this analysis on 21 August 2025. Patients with symptomatic unilateral or bilateral inguinal hernias aged 18 years or older were included. Exclusion criteria were recurrent inguinal hernias, previous open abdominal surgery at or below the umbilicus, liver disease defined by the presence of ascites, end-stage renal disease requiring dialysis, pregnancy, and emergency procedures.

### Study interventions

In the TEP procedure, the retromuscular space was dissected using a balloon dissector before a 12 mm port and two 5 mm ports were inserted below the umbilicus at the midline. Dissection was completed medially until full exposure of Cooper’s ligament was achieved and laterally until exposure of the psoas muscle was achieved. The hernia sac was separated from the spermatic cord in males or the round ligament in females and, after exclusion of a femoral hernia, a three-dimensional, monofilament mesh made of polyvinylidene fluoride (PVDF) measuring 10 × 15 cm or 12 × 17 cm and equipped with visual markers was inserted to cover the myopectineal orifice and attached to Cooper’s ligament (Dynamesh™ Endolap 3D visible). The mesh was secured using a cyanoacrylate-based tissue adhesive (Glubran 2^®^, GEMsrl, Italy).

In the rTAPP procedure, three 8 mm trocars were inserted and a robotic surgical system was docked (da Vinci Xi). The peritoneum was incised 4–5 cm above the internal inguinal ring. The dissection, hernia reduction, mesh-bed preparation, and mesh placement, including the use of the same mesh types, was performed identically to the TEP procedure. The peritoneal defect was closed using an absorbable barbed 3-0 suture (V-Loc™).

### Participating surgeons

This study required surgeons to be experienced in both rTAPP and TEP techniques. Eligibility criteria included having performed at least 30 of each procedure in the past year, as well as a documented history of over 50 advanced robotic procedures and broad experience in minimally invasive abdominal surgery.

A detailed description of the operative procedures and methodology can be found in the study protocol^10^. Patients with unilateral hernias were discharged on the day of surgery with paracetamol and metamizole for pain relief, unless indications for hospital admission were present such as urinary retention, pain out of proportion to the procedure, or social indications. Patients with bilateral hernias were admitted overnight as per local standards due to local insurance regulations.

### Objectives

It was hypothesized that rTAPP leads to a reduction of acute postoperative pain of 20% on a numeric rating scale (NRS) compared with TEP and would thus lead to earlier recovery^11,12^. The objective of this trial was therefore to investigate if rTAPP is associated with a decreased level of pain shortly after surgery when compared with laparoscopic TEP.

### Outcomes

The primary endpoint was postoperative pain while coughing 24 h after surgery, assessed using an NRS.

Secondary endpoints included NRS at rest 24 h after surgery as well as NRS at rest and while coughing after 7 days, 30 days, 6 months, and 12 months. Furthermore, hernia characteristics and procedural timings, length of stay, postoperative complications according to the Clavien–Dindo classification and the Comprehensive Complication Index (CCI), surgeon ergonomics assessed using the National Aeronautics and Space Administration (NASA) Task Load Index (TLX; range 0–100), short-form inguinal pain questionnaire (sf-IPQ; 0 = no pain/restriction—6 = maximum pain/restriction) results, European Quality of Life Five-Dimension Five-Level (EQ-5D-5L) questionnaire results, and the recurrence rate after 1 year assessed using an online questionnaire are reported^13–16^. Cost analysis was performed for procedural costs only, including material and operating room costs (costs per minute: 24.1 US dollars; as converted from Swiss francs, based on internal control data from St Claraspital Basel, Switzerland). Costs for length of stay, equipment acquisition, and maintenance were excluded, as those costs are highly variable amongst different healthcare institutions.

### Randomization and blinding

The patients were allocated to undergo TEP or rTAPP by block randomization, stratified for unilateral and bilateral hernias as well as for BMI (<30 and ≥30 kg/m^2^) to ensure the groups were comparable regarding these covariates. All patients and outcome assessors were blinded to the group allocation in the first 7 days after surgery. To enhance patient blinding, wound dressings for both surgical techniques were applied in both groups. The surgeons were not blinded and thus did not assess any outcomes within the first 7 days of surgery.

### Sample size determination and statistical considerations

Based on previous studies, a mean(s.d.) NRS of 4.4(1.7) was assumed 24 h after surgery in the control group (TEP)^17,18^. A 20% reduction in NRS for rTAPP compared with TEP was hypothesized. Given a level of significance of 0.05 and a power of 0.9, a sample size of 76 patients per group was calculated. With an estimated dropout rate of 20%, a minimum number of 91 patients was planned per group.

Treatment groups were analysed on an intention-to-treat (ITT) basis. Continuous data are presented as mean(s.d.) or median (interquartile range (i.q.r.)) and categorical data are presented as *n* (%). To compare normally distributed continuous data between two groups, Student’s *t*-test was used. Before applying this test, Levene’s test was performed to check for equal variances; if this assumption was not met, Welch’s *t* test was used instead. For continuous data that were not normally distributed, Wilcoxon’s rank-sum test served as a non-parametric alternative. For categorical data, the Mann–Whitney *U* test was used for ordinal data and Fisher’s exact test was used for nominal data. EQ-5D-5L outcome data were converted into a single preference-based index score, reflecting the utility value of the health state. Finally, Cohen’s d was calculated to determine the effect size, providing a measure of the difference between the two groups.

## Results

**Fig. 1:**
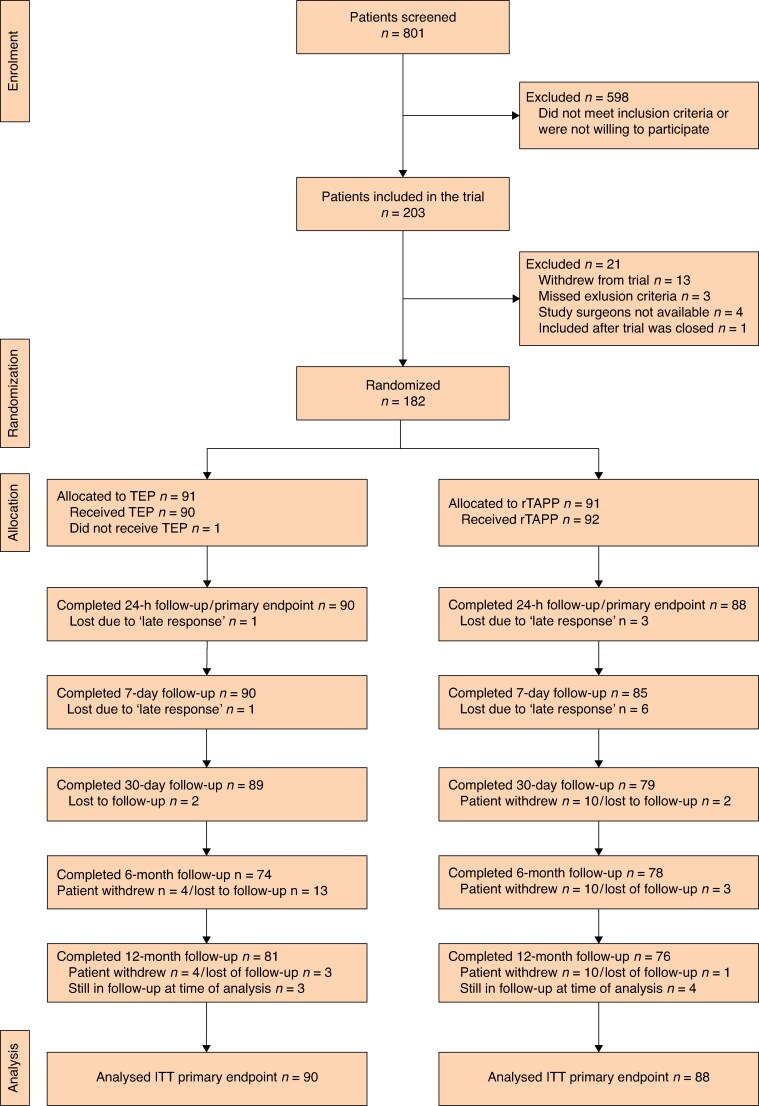
CONSORT diagram TEP, totally extraperitoneal repair; rTAPP, robotic transabdominal preperitoneal repair; ITT, intention to treat.

After screening for eligibility, a total of 203 patients were included in the trial. Twenty-one patients were excluded thereafter due to withdrawal while waiting for the procedure (13 patients), missed exclusion criteria during screening (3 patients), absence of qualified study surgeons (4 patients), and study termination due to reaching sample size (1 patient). Ultimately, 182 patients were randomized 1 : 1 to either undergo TEP or rTAPP. *Figure 1* shows the CONSORT flow diagram with detailed information on enrolment, allocation, analysis, and follow-up.

In total, 91 patients (93% male, mean(s.d.) age of 56.8(15.2) years, mean(s.d.) BMI of 24.8(3) kg/m^2^, and 22% with bilateral hernias) were allocated to the TEP group and 91 patients (95% male, mean(s.d.) age of 55.1(14.5) years, mean(s.d.) BMI of 24.6(2.9) kg/m^2^, and 21% with bilateral hernias) were allocated to the rTAPP group. Type of preoperative labour and ASA grade were comparable between the groups. There were more lateral hernias (78% *versus* 90%; *P* = 0.041) and more type III (>3 cm) hernias (39% *versus* 56%; *P* = 0.026) in the rTAPP group compared with the TEP group. A detailed description of the baseline characteristics is shown in *Table 1*.

**Table 1:** Baseline characteristics

	TEP (*n* = 91)	rTAPP (*n* = 91)	P*
**Sex**			>0.999
Male	85 (93)	86 (95)	
Female	6 (7)	5 (5)	
Age (years), mean(s.d.)	56.78(15.16)	55.12(14.53)	0.451
BMI (kg/m^2^), mean(s.d.)	24.84(3.01)	24.60(2.93)	0.582
**Type of labour**			0.920
Sedentary work	17 (18)	18 (20)	
Light work	23 (25)	18 (20)	
Medium work	13 (14)	17 (19)	
Heavy work	5 (6)	6 (7)	
Very heavy work	3 (3)	4 (4)	
Retired/unemployed	30 (33)	28 (31)	
**ASA grade**			0.462
I	9 (10)	14 (15)	
II	72 (79)	65 (71)	
III	10 (11)	12 (13)	
**EHS classification**			
Medial	49 (54)	53 (58)	0.654
Lateral	71 (78)	82 (90)	0.041
Femoral	5 (6)	11 (12)	0.189
I (<1.5 cm)	6 (7)	10 (11)	0.433
II (1.5–3 cm)	57 (63)	42 (46)	0.037
III (>3 cm)	35 (39)	51 (56)	0.026
**Localization**			>0.999
Unilateral	71 (78)	72 (79)	
Bilateral	20 (22)	19 (21)	

Values are *n* (%) unless otherwise indicated. *Fisher’s exact test or Welch’s two-sample *t* test. TEP, totally extraperitoneal repair; rTAPP, robotic transabdominal preperitoneal repair; EHS, European Hernia Society.

### Primary endpoint

**Fig. 2:**
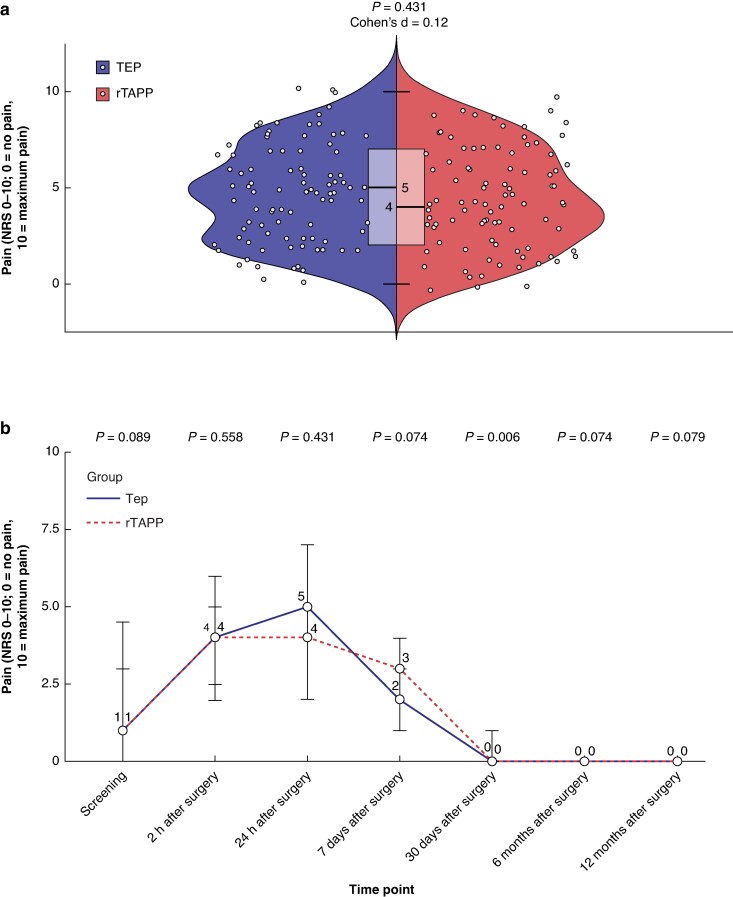
Primary outcome **a** Postoperative pain while coughing on an NRS 24 h after surgery. **b** Pain while coughing on an NRS at different time points. Values are presented as median (i.q.r.). NRS, numeric rating scale; i.q.r., interquartile range; TEP, totally extraperitoneal repair; rTAPP, robotic transabdominal preperitoneal repair.

For the analysis of the primary endpoint, 90 patients were available in the TEP group and 88 patients were available in the rTAPP group. Four patients did not respond within an acceptable time frame of less than 7 days and were therefore excluded from the analysis of the primary endpoint (*Fig. 1*). The median postoperative pain while coughing on an NRS 24 h after surgery was 5 (i.q.r. 2–7) after TEP and 4 (i.q.r. 2–7) after rTAPP (*P* = 0.431). The Cohen’s d of 0.12 indicates a small effect size. The individual NRS values for each patient and the nominal distribution are displayed in *Fig. 2a*.

### Secondary endpoints

The NRS while coughing at the time of screening was comparable between groups (*P* = 0.089; see *Fig. 2b*). Pain on an NRS at rest after 24 h was 2 (i.q.r. 1–3) in both groups (*P* = 0.964). From 7 days after surgery up to 12 months after surgery, pain on an NRS was comparable in both groups except after 30 days where NRS was higher in rTAPP, as demonstrated in *Fig. 2b*.

The mean(s.d.) operating time for unilateral hernias was 64.2(19.2) min for TEP and 80.3(20.9) min for rTAPP (*P* < 0.001) and the mean(s.d.) operating time for bilateral hernias was 102.7(23.8) min for TEP and 126.6(19.8) min for rTAPP (*P* = 0.002). In TEP, 10 × 15 cm meshes were more frequently inserted (63% in unilateral repair and 65% in bilateral repair) compared with rTAPP, where 12 × 17 cm meshes were more often applied (78% in unilateral repair and 50% in bilateral repair; *P* < 0.001 in unilateral and *P* = 0.011 in bilateral repair). The rate of day-case treatment was 87% for TEP and 82% for rTAPP (*P* = 0.489) in unilateral repair. Further data on perioperative outcomes are shown in *Table 2*.

**Table 2:** Perioperative details

	Unilateral			Bilateral		
	TEP (*n* = 71)	rTAPP (*n* = 72)	P*	TEP (*n* = 20)	rTAPP (*n* = 19)	P*
**Type of surgery**			0.489			>0.999
Day-case surgery	62 (87)	60 (83)		–	–	
Inpatient treatment	9 (13)	12 (17)		20 (100)	19 (100)	
Length of inpatient care (days), mean(s.d.)	1.75(1.39)	1.08(0.28)	0.216	1.30(0.66)	1.33(0.59)	0.870
Cut–suture time (min), mean(s.d.)	64.15(19.15)	80.34(20.93)	<0.001	102.65(23.76)	126.61(19.78)	0.002
Docking time for da Vinci Xi (min)†, mean(s.d.)	–	9.48(7.25)		6.00(–)	9.28(2.99)	
**Mesh size used**			<0.001			0.011
10 × 15cm	44 (63)	15 (21)		–	–	
12 × 17cm	26 (37)	57 (79)		–	–	
10 × 15 cm, both sides	–	–		13 (65)	3 (16)	
12 × 17 cm, both sides	–	–		4 (20)	10 (53)	
10 × 15 cm and 12 × 17 cm	–	–		3 (15)	6 (31)	

Values are *n* (%) unless otherwise indicated.*Fisher’s exact test or Welch’s two-sample *t* test. †TEP value due to ITT analysis. TEP, totally extraperitoneal repair; rTAPP, robotic transabdominal preperitoneal repair; ITT, intention to treat.

Ten (11%) postoperative complications occured after TEP and nine (10%) after rTAPP (*P* > 0.999). Whilst all complications after rTAPP were Clavien–Dindo grade II or lower, there was one Clavien–Dindo grade IIIb complication after TEP—a minimally invasive reintervention was required to evacuate a symptomatic retropubic postoperative haematoma that occurred due to wrongly applied bridging therapy in a patient needing therapeutic anticoagulation. There were no hernia recurrences after 6 months and one recurrence (1%) after 12 months in the TEP group (*P* > 0.999). Further information on postoperative outcomes is shown in *Table 3*.

**Table 3:** Postoperative outcomes

	TEP (*n* = 91)	rTAPP (*n* = 91)	P*
Patients with complication	10 (11)	9 (10)	>0.999
**Clavien–Dindo grade**			0.410
I	7 (8)	4 (4)	
II	2 (2)	5 (6)	
IIIb	1 (1)	0 (0)	
CCI, mean(s.d.)	1.54(5.16)	1.57(5.10)	0.965
**Recurrence**			
6 months	0 (0)	0 (0)	>0.999
12 months	1 (1)	0 (0)	>0.999
**NRS at rest, median (i.q.r.)**			
24 h	2.00 (1.00–3.00)	2.00 (1.00–3.00)	0.964
7 days	0.00 (0.00–1.00)	1.00 (0.00–1.00)	0.389
30 days	0.00 (0.00–0.00)	0.00 (0.00–1.00)	0.115
6 months	0.00 (0.00–0.00)	0.00 (0.00–0.00)	0.165
12 months	0.00 (0.00–0.00)	0.00 (0.00–0.00)	0.741
**NRS while coughing, median (i.q.r.)**			
24 h	5.00 (2.00–7.00)	4.00 (2.00–7.00)	0.431
7 days	2.00 (1.00–3.00)	3.00 (1.00–4.00)	0.074
30 days	0.00 (0.00–0.00)	0.00 (0.00–1.00)	0.006
6 months	0.00 (0.00–0.00)	0.00 (0.00–0.00)	0.074
12 months	0.00 (0.00–0.00)	0.00 (0.00–0.00)	0.079
**sf-IPQ, median (i.q.r.)**			
24 h	4.00 (3.00–7.00)	4.00 (3.00–6.00)	0.840
7 days	4.00 (3.00–6.00)	4.00 (3.00–6.00)	0.955
30 days	1.00 (1.00–3.00)	2.00 (1.00–3.00)	0.369
6 months	1.00 (1.00–1.00)	1.00 (1.00–1.00)	0.183
12 months	1.00 (1.00–1.00)	1.00 (1.00–1.00)	0.487

Values are *n* (%) unless otherwise indicated. *Fisher’s exact test, Welch’s two-sample *t* test, or Wilcoxon’s rank-sum test. TEP, totally extraperitoneal repair; rTAPP, robotic transabdominal preperitoneal repair; CCI, Comprehensive Complication Index; NR, numeric rating scale; SFsf-IPQ, short short-form inguinal pain questionnaire; TEP, Totally Extraperitoneal Plasty. rTAPP, robotic Transabdominal Preperitoneal Repair.

**Fig. 3:**
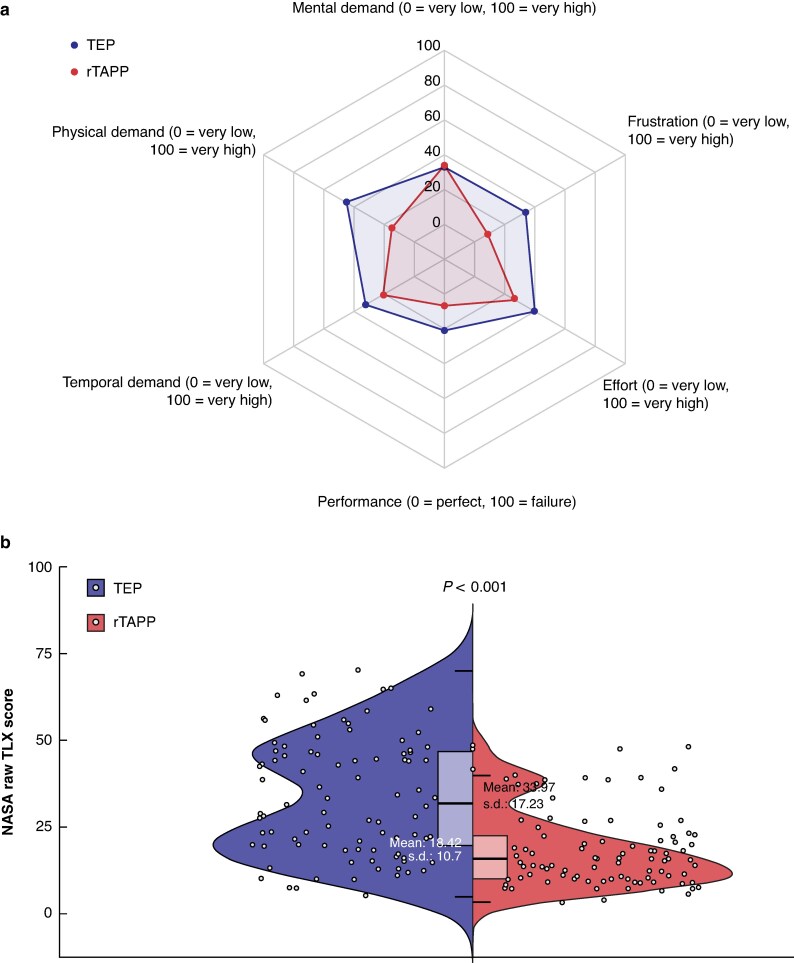
Surgeon task load, assessed using the NASA TLX (range 0–100) **a** Six subjective subscales. **b** NASA raw TLX score. NASA, National Aeronautics and Space Administration; TLX, Task Load Index; TEP, totally extraperitoneal repair; rTAPP, robotic transabdominal preperitoneal repair.

The mean(s.d.) NASA raw TLX score was 34.0(17.2) after TEP and 18.4(10.7) after rTAPP (*P* < 0.001). *Figure 3a* displays the six categories of the NASA TLX, showing a higher task load/demand for TEP compared with rTAPP for all of the categories, apart from mental demand. The TEP group exhibited greater variance in workload compared with the rTAPP group (*P* < 0.001, Levene’s test).

sf-IPQ after 24 h was 4 (i.q.r. 3–7) for TEP and 4 (i.q.r. 3–6) for rTAPP (*P* = 0.840). Seven days after surgery, sf-IPQ was 4 (i.q.r. 3–6) in both groups (*P* = 0.955). After 30 days, 6 months, and 12 months, sf-IPQ was low and similar between groups (values displayed in *Table 3*). Quality of life, assessed using the EQ-5D-5L questionnaire, was comparable at baseline (mean(s.d.) of 0.82(0.14) for TEP and 0.78(0.15) for rTAPP; *P* = 0.069), after 7 days (mean(s.d.) of 0.79(0.11) for TEP and 0.79(0.14) for rTAPP; *P* = 0.930), and after 12 months (0.96(0.09) for TEP and 0.95(0.08) for rTAPP; *P* = 0.617) (*Table S2*). The mean(s.d.) procedural costs for unilateral repair were 2732(458) US dollars for TEP and 3327(497) US dollars for rTAPP (*P* < 0.001). A detailed overview of the cost calculation is shown in *Table S1*.

## Discussion

This RCT was designed and powered to investigate for a difference in postoperative pain after laparoscopic TEP *versus* rTAPP. While it did not demonstrate superiority of rTAPP in terms of short-term postoperative pain, a higher proportion of patients undergoing rTAPP had concomitant lateral hernias that were detected and addressed, and larger meshes were implanted. Furthermore, a lower NASA raw TLX score signals higher satisfaction and comfort of surgeons with rTAPP compared with TEP.

Postoperative pain 24 h and up to 12 months after surgery in patients undergoing TEP *versus* rTAPP for inguinal hernia repair were similar, except for a clinically negligible difference in favour of TEP at 30 days post-surgery. When comparing pain and postoperative functional ability, it is worth noting the main technical differences between TEP and rTAPP: in TEP there is no need for routine peritoneal defect closure and the mesh fixation is atraumatic using glue, whereas rTAPP includes suturing of the peritoneal defect and fixation of mesh with resorbable sutures. These technical aspects may theoretically influence postoperative pain. However, a systematic review and sequential trial analysis of RCTs including 1359 patients did not show any difference in postoperative pain or chronic pain between TEP and TAPP, thus the comparison of laparoscopic TEP and rTAPP is reasonable and justified^19^. Furthermore, conventional TEP has been the standard procedure in the authors’ institution for several years and was thus chosen as the comparator to rTAPP to avoid learning-curve bias.

The findings of the present study are consistent with the results of the RIVAL trial^4^, an RCT comparing laparoscopic TAPP with rTAPP, which found no difference in pain and perioperative outcomes up to 30 days after surgery; in addition, the operating time was longer in both the present RCT and the RIVAL trial when a robot was used. Conversely, the recently published ROLAIS trial^5^ reported a reduced complication rate and a shorter operating time for rTAPP compared with conventional TAPP; however, an analysis of postoperative pain was not conducted. Furthermore, a reduced stress response, assessed through the measurement of postoperative C-reactive protein (CRP) and interleukin 6 (IL-6) levels, was reported in the ROLAIS trial^5^; the clinical significance of this finding, however, remains debatable. Therefore, current high-quality evidence demonstrates no superiority of robotic assistance in minimally invasive inguinal hernia repair in terms of postoperative pain or functional impairment.

Although patients were randomized and stratified according to BMI, there were important differences in the hernia characteristics, with larger and more concomitant lateral hernias in the rTAPP group. On one hand, these differences in baseline characteristics could explain the insertion of larger meshes and the longer operating time in the rTAPP group. On the other hand, it could be assumed that, due to better exposure in rTAPP, a higher rate of concomitant lateral hernias was detected, while the proportion of medial hernias was similar. Given the easier preparation for rTAPP, a space for a larger mesh can be established, while still reducing surgeon workload compared with TEP. Further reasons for the longer operating time in rTAPP, however, may be the need for robot docking and the inherent differences between rTAPP and TEP, with suture mesh fixation and peritoneal suturing necessary for rTAPP. A longer operating time for rTAPP was also reported in other studies that compared it with conventional laparoscopic repair^6,7^.

The length of stay in the present study is mainly driven by local health regulations. Bilateral repairs and unilateral repairs in co-morbid patients are routinely performed in a day-case setting, whereas healthy patients undergoing unilateral repairs are discharged on the day of the procedure. The rate of day-case treatment success, however, was comparable to or even higher than that reported in recently published studies^20,21^.

The overall complication rate was low and there were no differences between groups. There was only one recurrence in 182 patients after 1 year, which occurred in the TEP group. An increased rate of surgical-site infections after robotic inguinal hernia repair, previously demonstrated in a systematic review and meta-analysis comparing robotic with laparoscopic inguinal hernia repair^22^, was not observed. The recently published ROLAIS trial^5^, comparing rTAPP with conventional TAPP, showed a lower complication rate for rTAPP (with the complications being mainly haematomas and urinary retention), whereas the rate of reinterventions was comparable between the groups. In the present study, quality of life, assessed using the EQ-5D-5L questionnaire, was comparable between the groups at baseline and after surgery, showing an expected decrease in the early postoperative phase followed by stabilization in the later postoperative phase in both groups. Thus, there seems to be no benefit regarding quality of life when comparing rTAPP *versus* conventional TEP.

Regarding surgeon task load, the present study indicates a lower NASA raw TLX score for the rTAPP procedure. Apart from mental demand, rTAPP showed more favourable scores for all of the categories of the index, even for temporal demand, despite longer operating times. The RIVAL trial reported conflicting NASA TLX scores, where rTAPP was rated to have a higher overall surgeon task load compared with conventional TAPP^4^. It should be noted that the TEP procedure may, in general, be associated with a higher overall workload compared with (laparoscopic) TAPP. The data from the present study indicate that the workload distribution for rTAPP shows less variance compared with TEP, for which some cases result in a very high workload and others result in a low workload (*P* < 0.001, Levene’s test). This finding is also consistent with the authors’ subjective impression of the two operative techniques (*Fig. 3b*). Even though the NASA TLX involves fundamentally subjective assessment, it is a validated tool and it can be concluded that, overall, rTAPP results in a reduced surgeon workload. Given the overall low workload associated with rTAPP, it could be argued that it may serve as a potential entry point for surgeons into robotic hernia surgery.

Procedural costs were lower for TEP, mainly due to the shorter operating time. Cost analyses, however, can be highly variable amongst different healthcare institutions and for different surgeons, as, for example, many surgeons do not use a structural ballon for dissection or avoid mesh fixation with glue in TEP. Costs for rTAPP instruments are high, but are likely to become more affordable upon widespread adoption of robotic surgery in the near future.

This study has some limitations. First, it is a single-centre study and therefore the results are not generally applicable to other healthcare institutions. Second, the sample size calculation was based on postoperative pain as the primary outcome and thus the sample size may be insufficient to identify differences in other outcomes such as perioperative complications or recurrence rates. Furthermore, the follow-up duration is limited to 1 year, which is likely insufficient to report on recurrence rates. Lastly, a potential source of bias in the present study is the difference in surgical expertise between the two approaches. While a minimum case volume was set to mitigate inexperience, all participating surgeons were inherently more proficient in TEP due to it being the standard surgical approach in the authors’ institution. The literature on the rTAPP learning curve is inconsistent, with requirements ranging from >25 total cases in the RIVAL trial^4^ to >100 rTAPP cases in the ROLAIS trial^5^. The requirement of at least 30 TEP/rTAPP cases within the past year in the present study falls within this range, but is significantly lower than some proposed standards. Consequently, it is possible that some of the rTAPP procedures were performed by surgeons who had not yet completed the learning curve.

There is no superiority of rTAPP compared with laparoscopic TEP with respect to postoperative pain. Morbidity and recurrence rates are low and comparable for both procedures. rTAPP is associated with a reduced surgeon workload at the expense of a longer operating time. Widespread adoption of rTAPP remains debatable considering these findings. Future studies should investigate the impact of surgeon experience on rTAPP outcomes and the role of surgeon ergonomics and workload in robotic surgery warrants greater consideration in future trials.

## Supplementary Material

znaf283_Supplementary_Data

## Data Availability

The data, analytic methods, and study materials that support the findings of this study are available from the corresponding author upon request.
