# Supplementary material for: Robotic versus laparoscopic minimally invasive inguinal hernia repair: randomized clinical trial (the ROGER trial)
Source: Br J Surg. 2026 Jan 9;113(1):znaf283. doi: 10.1093/bjs/znaf283 (PMC12785885; doi:10.1093/bjs/znaf283)
Supplement: znaf283_Supplementary_Data [file znaf283_supplementary_data.zip › Supplementary_material.docx]

**Robotic versus laparoscopic Minimally-Invasive Inguinal Hernia Repair: a randomized clinical trial (ROGER-Trial)**

Fiorenzo V. Angehrn1*+, Julian Süsstrunk1+, Romano Schneider1, Kaspar Baltzer1, Beat P. Müller1, Johannes Baur1+, Daniel C Steinemann1+

+: Contributed equally

1 Clarunis, Department of Visceral Surgery, University Digestive Health Care Center, St. Clara Hospital and University Hospital Basel, Basel, Switzerland

**Corresponding author:**
Fiorenzo V. Angehrn, Clarunis, Department of Visceral Surgery, University Digestive Health Care Center, St. Clara Hospital and University Hospital Basel, Basel, Switzerland **ORCID ID 0000-0002-5450-3100**

**Supplementary Materials - Index**

| **Supplementary Methods** |  |
| --- | --- |
| - |  |
| **Supplementary Results** |  |
| - |  |
| **Supplementary Appendixes** |  |
| Clinical Study Protocol | *pag. 5* |
| **Supplementary Figures and Tables** |  |
| **Table S1:** Overview of procedural costs in US dollars. TEP: Totally Extraperitoneal Plasty. rTAPP: robotic Transabdominal Preperitoneal Repair. | *pag. 51* |
| **Table S2:** European Quality of Life 5 Dimensions 5 Level Version (EQ-5D-5L) questionnaire results. TEP: Totally Extraperitoneal Plasty. rTAPP: robotic Transabdominal Preperitoneal Repair. | *pag. 52* |
| **References** |  |
| - |  |

**Supplementary Methods**

**-**

**Supplementary Results**

**-**

**Supplementary Appendixes**

**Clinical Study Protocol**

**Change history**

| Version Nr | Version date | Modified without version change | Description, comments | Control |
| --- | --- | --- | --- | --- |
| 1d | 23.06.2021 |  | Initial version | PG |
| 1e | 10.09.2021 |  | Revision 1 | PG |

Robotic versus conventional minimal-invasive inguinal hernia repair – a prospective, randomized and blinded clinical trial (ROGER-RCT)

Clinical Study Protocol

Prospective, randomized, blinded clinical trial, single center, Basel Switzerland

SHORT TITLE: ROGER-RCT

| Study Type: | Patient and assessor blinded randomized controlled clinical trial |
| --- | --- |
| Study Categorisation: | Risk category A (KlinV Art. 61) |
| Study Registration: | The study will be registered after ethical approval in www.clinicaltrials.gov |
| Study Identifier: | Not yet applicable |
| Principal Investigator: | Fiorenzo Angehrn, MD  Consultant Clarunis  FMH Chirurgie  Clarunis AG  Postfach, 4002 Basel  fiorenzo.angehrn@clarunis.ch  Tel. 061 777 73 17  Fiorenzo.angehrn@clarunis.ch  Phone +41 61 777 73 17 |
| Investigational Product: | Surgical robot (da Vinci Xi, Intuitive) |
| Protocol Version and Date: | Version 1e, 10.09.2021 |

CONFIDENTIAL

The information contained in this document is confidential and the property of the “principal investigator”. The information may not - in full or in part - be transmitted, reproduced, published, or disclosed to others than the applicable Competent Ethics Committee(s) and Regulatory Authority(ies) without prior written authorisation from the sponsor except to the extent necessary to obtain informed consent from those who will participate in the study.

Signature Page(s)

Note: Add more lines, functions and pages if relevant, e.g. for trial statistician, if relevant or protocol contributors

| Study number | n.a. |
| --- | --- |
| Study Title | Robotic versus conventional minimal-invasive inguinal hernia repair – a prospective, randomized and blinded clinical trial (ROGER-RCT) |

The Sponsor-Investigator and trial statistician have approved the protocol version 1e dated 10.09.2021 and confirm hereby to conduct the study according to the protocol, current version of the World Medical Association Declaration of Helsinki, the ICH-GCP guidelines and the local legally applicable requirements.

Sponsor-Investigator:

Fiorenzo Angehrn, Consultant Clarunis

| Place/Date |  | Signature |
| --- | --- | --- |

Local Principal Investigator at study site*:

I have read and understood this trial protocol and agree to conduct the trial as set out in this study protocol, the current version of the World Medical Association Declaration of Helsinki, ICH-GCP guidelines and the local legally applicable requirements.

| Site | Clarunis, St. Claraspital AG, Kleinriehenstrasse 30, 4058 Basel |
| --- | --- |
| Principal investigator | Fiorenzo Angehrn, Consultant Clarunis |

| Place/Date |  | Signature |
| --- | --- | --- |

**Note:* In multicentre studies, this page must be individually signed by all participating Local Principal Investigators.

**Table of Contents**

Study synopsis 8

Abbreviations 12

Summary of the revision History in case of amendments 14

Study schedule 14

1. STUDY ADMINISTRATIVE STRUCTURE 15

1.1 Sponsor, Sponsor-Investigator 15

1.2 Principal Investigator(s) 15

1.3 Statistician ("Biostatistician") 16

1.4 Laboratory 16

1.5 Monitoring institution 16

1.6 Data Safety Monitoring Committee 16

1.7 Any other relevant Committee, Person, Organisation, Institution 16

2. ETHICAL AND REGULATORY ASPECTS 17

2.1 Study registration 17

2.2 Categorisation of study 17

2.3 Competent Ethics Committee (CEC) 17

2.4 Competent Authorities (CA) 17

2.5 Ethical Conduct of the Study 17

2.6 Declaration of interest 18

2.7 Patient Information and Informed Consent 18

2.8 Participant privacy and confidentiality 18

2.9 Early termination of the study 18

2.10 Protocol amendments 19

3. Background and Rationale 20

3.1 Background and Rationale 20

3.2 Investigational Product (treatment) and Indication 21

3.3 Preclinical Evidence 21

3.4 Clinical Evidence to Date 21

3.5 Rationale for the dosage, route, regimen 21

3.6 Explanation for choice of comparator (or placebo) 21

3.7 Risks / Benefits 21

3.8 Justification of choice of study population 21

4. STUDY OBJECTIVES 23

4.1 Overall Objective 23

4.2 Primary Objective 23

4.3 Secondary Objectives 23

4.4 Safety Objectives 23

5. STUDY OUTCOMES 24

5.1 Primary Outcome 24

5.2 Secondary Outcomes 24

5.3 Safety Outcomes 24

6. STUDY DESIGN 26

6.1 General study design and justification of design 26

6.2 Methods of minimising bias 26

6.2.1 Randomisation 26

6.2.2 Blinding procedures 26

6.2.3 Other methods of minimising bias 26

6.3 Unblinding Procedures (Code break) 27

7. STUDY POPULATION 28

7.1 Eligibility criteria 28

7.2 Recruitment and screening 28

7.3 Assignment to study groups 28

7.4 Criteria for withdrawal / discontinuation of participants 28

8. STUDY INTERVENTION 29

8.1 Identity of Investigational Products 29

8.1.1 Experimental Intervention 29

8.1.2 Control Intervention (standard/routine/comparator treatment) 29

8.1.3 Packaging, Labelling and Supply (re-supply) 29

8.1.4 Storage Conditions 30

8.2 Administration of experimental and control interventions 30

8.2.1 Experimental Intervention 30

8.2.2 Control Intervention 30

8.3 Dose modifications 30

8.4 Compliance with study intervention 30

8.5 Data Collection and Follow-up for withdrawn participants 30

8.6 Trial specific preventive measures 30

8.7 Concomitant Interventions (treatments) 31

8.8 Study Drug Accountability 31

8.9 Return or Destruction of Study Drug 31

9. STUDY ASSESSMENTS 32

9.1 Study flow chart(s) / table of study procedures and assessments 32

9.2 Assessments of outcomes 32

9.2.1 Assessment of primary outcome 32

9.2.2 Assessment of secondary outcomes 33

9.2.3 Assessment of other outcomes of interest 33

9.2.4 Assessment of safety outcomes 33

9.2.5 Assessments in participants who prematurely stop the study 33

9.3 Procedures at each visit 33

9.3.1 Screening visit 33

9.3.2 Admission time 34

9.3.3 In the operating room, before incision 34

9.3.4 Surgical intervention 34

9.3.5 Early postoperative (up to 24h postop) 34

9.3.6 Follow-up (24h – 12 months postop) 34

10. SAFETY 35

10.1 Medical Device Category A studies 35

10.1.1 Definition and Assessment of safety related events 35

10.1.2 Reporting of Safety related events 35

11. STATISTICAL METHODS 36

11.1 Hypothesis 36

11.2 Determination of Sample Size 36

11.3 Statistical criteria of termination of trial 36

11.4 Planned Analyses 36

11.4.1 Datasets to be analysed, analysis populations 37

11.4.2 Primary Analysis 37

11.4.3 Secondary Analyses 37

11.4.4 Interim analyses 37

11.4.5 Safety analysis 37

11.4.6 Deviation(s) from the original statistical plan 37

11.5 Handling of missing data and drop-outs 37

12. QUALITY ASSURANCE AND CONTROL 38

12.1 Data handling and record keeping / archiving 38

12.1.1 Case Report Forms 38

12.1.2 Specification of source documents 38

12.1.3 Record keeping / archiving 38

12.2 Data management 38

12.2.1 Data Management System 38

12.2.2 Data security, access and back-up 39

12.2.3 Analysis and archiving 39

12.2.4 Electronic and central data validation 39

12.3 Monitoring 39

12.4 Audits and Inspections 39

12.5 Confidentiality, Data Protection 39

12.6 Storage of biological material and related health data 39

13. PUBLICATION AND DISSEMINATION POLICY 40

14. FUNDING AND SUPPORT 40

14.1 Funding 40

14.2 Other Support 40

15. INSURANCE 40

16. REFERENCES 41

17. APPENDICES 44

Study synopsis

Provide a structured synopsis containing all important information, preferably in tabular view:

| Sponsor / Sponsor-Investigator | Dr. med. Fiorenzo Angehrn  Oberarzt Clarunis  FMH Chirurgie  Clarunis AG  Fiorenzo.angehrn@clarunis.ch  Tel. 061 777 73 17 |
| --- | --- |
| Study Title: | Robotic versus conventional minimal-invasive inguinal hernia repair – a prospective, randomized and blinded clinical trial (ROGER-RCT) |
| Short Title / Study ID: | ROGER-RCT |
| Protocol Version and Date: | Version 1e, 10.09.2021 |
| Trial registration: | The study will be registered after ethical approval in www.clinicaltrials.gov and www.kofam.ch |
| Study category and Rationale | Risk category A (KlinV Art. 61).  In this study a well-known surgical robot (da Vinci) will be used to perform an inguinal hernia repair. This procedure is already carried out with and without the da Vinci everyday worldwide and in Switzerland. The da Vinci is authorized in Switzerland and is applied in accordance with the prescribing information. |
| Clinical Phase: | Clinical study phase: 4 |
| Background and Rationale: | The aim of this study is to evaluate whether a robot-assisted inguinal hernia repair can reduce the postoperative pain and enhance recovery compared to the conventional laparoscopic approach.  All included hernia patients will undergo surgery under general anaesthesia. According to current guidelines, minimal invasive procedures such as transabdominal preperitoneal hernia repair (TAPP) or total extraperitoneal endoscopic hernioplasty (TEP) are the preferred way. Patients will be randomized to either the experimental group robotic transabdominal preperitoneal hernia repair (rTAPP) or the control group conventional total extraperitoneal endoscopic hernioplasty (TEP). According to literature, TAPP and TEP in conventional technique show comparable results. However, in rTAPP fixation of the mesh using tackers may be avoided, the dissection is more precise and allows better preservation of nerve structures. Thus, a reduction of postoperative pain is assumed. |
| Objective(s): | With this randomized and blinded study we compare rTAPP to conventional TEP with short-term pain as primary outcome (numeric rating scale (NRS) surgery). Secondary endpoints include comparison of pain in a longer course (short-form inguinal pain questionnaire (sf-IPQ)), quality of life / health status (Baseline Short Form-12 (SF-12), Carolinas Comfort Scale (CCS)), complications (Comprehensive Complication Index - CCI), rate of recurrence, economic impact in terms of costs of surgery per patient, for the institution, the sick leave and the cost-effectiveness of health intervention (SF-6D, EQ-5D, ICECAP-O). Also included are ergonomics for the surgeon (NASA TLX and specific Questionnaire). |
| Outcome(s): | Primary endpoint:  - Pain at coughing 24 hours after surgery measured on a numeric rating scale (NRS 0-10) while coughing.  Secondary endpoints include:  - NRS score 2 hours, 7 days and 30 days, 6 and 12 months after surgery.  - Inguinal pain questionnaire (sf-IPQ) 24 hours, 7 days and 30 days, 6 and 12 months after surgery  - EQ-5D-5L 24 hours, 7 days and 30 days, 6 and 12 months after surgery  - SF-6D 24 hours, 7 days and 30 days, 6 and 12 months after surgery  - ICECAP-O 24 hours, 7 days and 30 days, 6 and 12 months after surgery  - Intraoperative complications  - Amount of perioperative pain medication  - Procedure time  - Time in the OR block  - For day surgery hours in outpatient clinic until discharge  - For hospitalized patients postoperative stay in days  - Prescribed and actually taken pain medication postoperative 24 hours, 7 days and 30 days, 6 and 12 months after surgery  - Postoperative morbidity classified according to the Dindo-Clavien classification and scored according to the Comprehensive complication index (CCI) up to 30 days after surgery.  - Recurrence rate 6 and 12 months postoperative. Recurrences are excluded clinically and by sonography by the operating surgeon (face-to-face visit).  - Quality of life (SF-12) 30 days, 6 months, and 12 months postoperative.  - Carolinas Comfort Scale (CCS) 30 days, 6 months, and 12 months postoperative.  - Ergonomics for the surgeon measured by NASA TLX and specific questionnaire.  - Experience of the surgeons in rTAPP and/or TEP (performed surgeries in the last 12 months)  - Costs for surgery per patient according to the accounting department (costs for care, operating room, anesthesia, consumables)  - Sick leave (days until resumption of work or days until resumption of activities of daily life, and estimated sick leave by patient).  - Costs for sick leave (days multiplied by average daily costs of sick leave in Switzerland according to the Swiss National Accident Insurance Fund (SUVA)).  - Type of labor including the relative activity level (sedentary work, light work, medium work, heavy work, very heavy work, retired/unemployed) according to US Code of Federal Regulations § 404.1567 Physical exertion requirements. |
| Study design: | Patient and assessor blinded randomized controlled trial. |
| Inclusion / Exclusion criteria: | Patients:  The study population will be patients with a given surgical indication for a unilateral or bilateral inguinal hernia.  Inclusion criteria  • Patients older than 18 years of age and able to understand and give their informed consent for the study.  • Primary unilateral or bilateral hernia  Exclusion criteria  • Recurrent hernia  • with previous open abdominal surgery below the umbilicus  • need of an open inguinal hernia repair (patient’s preference, unable to undergo general anesthesia, unable to tolerate pneumoperitoneum)  • liver disease defined by the presence of ascites  • end-stage renal disease requiring dialysis  • unable to give informed consent  • need of an emergency surgery  • pregnancy  Criteria for participating surgeons  • Each participating surgeon has performed >50 advanced robotic procedures (e.g. colorectal surgery, esophageal surgery, fundoplication, hiatal hernia repair) before study start.  • Each participating surgeon has performed >30 rTAPP within the last 12 months.  • Each particating surgeons has performed >30 TEP within the last 12 months.  • All surgeons participating in the study will perform rTAPP as well as TEP.  • It is planned that 5 surgeons will perform the surgeries.  • These surgeons have broad experience in minimal invasive abdominal surgery. |
| Measurements and procedures: | All patients satisfying the previous criteria will be included in the study and will undergo several steps:  A preoperative consultation with:  - collection of demographic data (age, sex, principal diagnosis, preoperative diseases, type and percentage of work).  - Daily pain and quality of life (through questionnaires).  - Elucidation of the study and submission of the informed consent  In the admission day, the informed consent will be collected.  After preoperative consultation, randomized selection in the rTAPP vs TEP (Patient and assessor blinded).  Patients follow up:  - Post-operative controls 2h, 24 hours (by phone call)  - Follow up day 7, 30 by phone call; after 6 and 12 months face-to-face (NRS, short-form inguinal pain questionnaire (sf-IPQ)), quality of life / health status (Baseline Short Form-12 (SF-12), Carolinas Comfort Scale (CCS)), complications (Comprehensive Complication Index - CCI), rate of recurrence, , economic impact in terms of costs of surgery per patient, for the institution, the sick leave and the cost-effectiveness of health intervention (SF-6D, EQ-5D, ICECAP-O)).  Surgeons ergonomics:  - NASA-TLX (Task Load Index and specific questionnaire postoperatively day 0 |
| Study Product / Intervention: | In the intervention group the da Vinci robot will be used to perform a transabdominal preperitoneal hernia repair (rTAPP) |
| Control Intervention (if applicable): | The control group will receive a conventional total extraperitoneal endoscopic hernioplasty (TEP). |
| Number of Participants with Rationale: | An NRS of 4.37 ± 1.66 24 hours after surgery at coughing in the control group is assumed. The study hypothesis is a 20% reduction of pain at coughing on postoperative day 1 (at 24 hours). Given a level of significance of 0.05 and a power of 0.90 a sample size of 76 patient per group are needed. A drop out rate of 20% is estimated. Therefore we plan to include a total of 182 patients. |
| Study Duration: | We estimate to screen and include patients over 1 years given a number of 182 to be included. The last follow-up will take place 12 month post-surgery of the last included patients. Overall the study’s end is estimated therefore 2 years after inclusion of the first patient. |
| Study Schedule: | Month Year of First-Participant-In (planned): 01/22  Month Year of Last-Participant-Out (planned 12/23) |
| Investigator(s): | Dr. med. Fiorenzo Angehrn  Oberarzt Clarunis  Clarunis AG  Postfach, 4002 Basel  fiorenzo.angehrn@clarunis.ch  Tel. 061 777 73 17  PD Dr. med. Daniel Steinemann  Leitender Arzt  FMH Chirurgie, EBSQ Koloproktologie, Schwerpunkt Viszeralchirurgie  Clarunis AG  Postfach, 4002 Basel  daniel.steinemann@clarunis.ch  PD Dr. med. Martin Bolli  Stv. Chefarzt  FMH Chirurgie, Schwerpunkt Viszeralchirurgie  Clarunis AG  Postfach, 4002 Basel  martin.bolli@clarunis.ch |
| Study Centre(s): | Single-Center in Switzerland (Clarunis, Claraspital) |
| Statistical Considerations: | Analysis is performed on an intention-to-treat basis with all patients in the group to which they were allocated. Data were entered in a study database and analyzed using SPSS. Continuous variables are depicted as means with the 95 percent confidence interval. The Mann-Whitney U-test is used to compare continuous data, and the Fisher’s exact test for analysis of categorical variables. Multiple group comparisons of continuous variables are performed using a two-sided ANOVA test.  Pearson’s r-correlation will be used to correlate the primary and secondary outcome with surgical experience as well as patient’s characterics such as BMI, uni- vs bilateral hernia, sex, and age. |
| GCP Statement: | This study will be conducted in compliance with the protocol, the current version of the Declaration of Helsinki, ICH-GCP, as well as all national legal and regulatory requirements. |

Abbreviations

Provide a list of abbreviations used on the protocol - to be completed

| AE | | Adverse Event | | |
| --- | --- | --- | --- | --- |
| CA | | Competent Authority (e.g. Swissmedic) | | |
| CEC | | Competent Ethics Committee | | |
| CRF | | Case Report Form | | |
| ClinO | | Ordinance on Clinical Trials in Human Research *(in German: KlinV, in French: OClin)* | | |
| eCRF | | Electronic Case Report Form | | |
| CTCAE | | Common terminology criteria for adverse events | | |
| DSUR | | Development safety update report | | |
| GCP | | Good Clinical Practice | | |
| IB | | Investigator’s Brochure | | |
| Ho | | Null hypothesis | | |
| H1 | | Alternative hypothesis | | |
| HFG | | Humanforschungsgesetz (Law on human research) | | |
| HMG | | Heilmittelgesetz | | |
| HRA | | Federal Act on Research involving Human Beings | | |
| IMP | | Investigational Medicinal Product | | |
| IIT | | Investigator-initiated Trial | | |
| ISO | | International Organisation for Standardisation | | |
| ITT | | Intention to treat | | |
| KlinV | | Verordnung über klinische Versuche in der Humanforschung *(in English: ClinO, in French OClin)* | | |
| LPTh | | Loi sur les produits thérapeutiques | | |
| LRH | | Loi fédérale relative à la recherche sur l’être humain | | |
| MD | | Medical Device | | |
| OClin | | Ordonnance sur les essais cliniques dans le cadre de la recherche sur l'être humain *(in German : KlinV, in English : ClinO)* | | |
| PI | | Principal Investigator | | |
| SDV | | Source Data Verification | | |
| SOP | | Standard Operating Procedure | | |
| SPC | | Summary of product characteristics | | |
| SUSAR | | Suspected Unexpected Serious Adverse Reaction | | |
| TMF | | Trial Master File | | |
| TAPP | | Transabdominal preperitoneal hernia repair | | |
| TEP | | Total extraperitoneal endoscopic hernioplasty | | |
| rTAPP | | Robotic transabdominal preperitoneal hernia repair | | |
| NRS | | Numeric rating scale | | |
|  | |  |  |  |

Summary of the revision History in case of amendments

| **Version Nr, Version Date** | **Chapter** | **Description of change** | **Reason for the change** |
| --- | --- | --- | --- |
|  |  |  |  |

Study schedule

Insert a flow chart (graphic) or tabular listing of schedule of events and assessments and procedures of the study (an example is provided below, amend and expand according to the specific study). To be repeated in 9.1.

|  | Baseline | Day 0, 2h postop | 24h postop | 7d postop | 30d postop | 6 months | 12 months |
| --- | --- | --- | --- | --- | --- | --- | --- |
| Assessment | Face-to-face | Face-to-face | Telephone | Telephone | Telephone | Face-to-face | Face-to-face |
| NRS (primary endpoint) | + |  | + |  |  |  |  |
| NRS |  | + |  | + | + | + | + |
| sf-IPQ | + |  | + | + | + | + | + |
| EQ-5D-5L | + |  | + | + | + | + | + |
| ICECAP-O | + |  | + | + | + | + | + |
| morbidity |  |  |  |  | + |  |  |
| Intraoperative complications |  | + |  |  |  |  |  |
| Perioperative  pain medication |  | + |  |  |  |  |  |
| OR and procedure time |  | + |  |  |  |  |  |
| Hospital stay |  | + |  |  |  |  |  |
| Pain medication (prescribed and intake) |  | + | + | + | + | + | + |
| Recurrence |  |  |  |  |  | + | + |
| SF-12 | + |  |  |  | + | + | + |
| CCS | + |  |  |  | + | + | + |
| SF-6D | + |  | + | + | + | + | + |
| NASA TLX | + | + |  |  |  |  |  |
| specific questionnaire | + | + |  |  |  |  |  |
| Costs for surgery |  |  |  |  | + |  |  |
| Sick leave |  |  |  |  | + |  |  |
| Costs for sick leave |  |  |  |  | + |  |  |
| Type of Labor | + |  |  |  | + | + | + |

# STUDY ADMINISTRATIVE STRUCTURE

Any committee(s) to be formed should be mentioned here (e.g., safety committees, data monitoring committees, etc.). Subsections may be expanded if necessary but shall not be deleted if not relevant.

Describe a solution, if not all personnel involved are determined at this stage and may be referred to other documents than the protocol.

Provide complete contact details (address, phone, e-mail) of all individuals or groups/committees and their composition, roles, and responsibilities overseeing the trial (e.g. Sponsor, PI, statistician, monitor, coordinator, any committee, data management team, and other individuals or groups, laboratories if applicable).

## Sponsor, Sponsor-Investigator

Dr. med. Fiorenzo Angehrn

Oberarzt Clarunis

Clarunis AG

Postfach, 4002 Basel

fiorenzo.angehrn@clarunis.ch

Tel. 061 777 73 17

The sponsor-investigator is responsible for the study design, collection, management, analysis and interpretation of data. He will write a report of this study intended to be published in a peer-reviewed journal.

## Principal Investigator(s)

Dr. med. Fiorenzo Angehrn

Oberarzt Clarunis

Clarunis AG

Postfach, 4002 Basel

fiorenzo.angehrn@clarunis.ch

Tel. 061 777 73 17

PD Dr. med. Daniel Steinemann

Leitender Arzt

FMH Chirurgie, EBSQ Koloproktologie, Schwerpunkt Viszeralchirurgie

Clarunis AG

Postfach, 4002 Basel

daniel.steinemann@clarunis.ch

PD Dr. med. Martin Bolli

Stv. Chefarzt

FMH Chirurgie, Schwerpunkt Viszeralchirurgie

Clarunis AG

Postfach, 4002 Basel

martin.bolli@clarunis.ch

# ETHICAL AND REGULATORY ASPECTS

The decision of the CEC and Swissmedic/foreign competent authority concerning the conduct of the study will be made in writing to the Sponsor-Investigator before commencement of this study. The clinical study can only begin once approval from all required authorities has been received. Any additional requirements imposed by the authorities shall be implemented.

## Study registration

After ethical approval, the study will be register in [www.clinicaltrials.gov](http://www.clinicaltrials.gov)and [www.kofam.ch](http://www.kofam.ch).

## Categorisation of study

The clinical trial come under Category A according to KlinV Art. 61 because the performed surgeries in both groups are well established and routinely used.

## Competent Ethics Committee (CEC)

The principle investigator will ensure that approval of the following responsible Ethics Committeee is requested:

Ethikkommission Nordwest- und Zentralschweiz

Adverse events (AE) are any unfavourable and unintended sign, symptom, or disease temporarily associated with the study participation. An adverse event is considered a serious adverse event (SAE) if it results in any of the following outcomes: death, life-threatening condition, inpatient hospitalization or prolongation of existing hospitalization, persistent or significant incapacitiy or substantial disruption of ability to conduct normal life functions. All AE and SAE are documented in the CRF. SAE, changes in research activity and all unanticipated problems involving risks to humans will be reported to the Ethics Committee within 15 days. The Ethics Committee will receive an announcement of regular study termination within 90 days. An eventual premature termination or interruption report will be announced within 15 days. A final study report will be sent to Ethics Committee within one year after termination of the study.

No changes are made to the protocol without prior sponsor and Ethics Committee approval, except where necessary to eliminate apparent immediate hazards to study participants.

## Competent Authorities (CA)

No Competent Authorities approval is necessary as the Study has a Category A (KlinV Art. 61) risk.

## Ethical Conduct of the Study

The study will be carried out in accordance to the protocol and with principles enunciated in the current version of the Declaration of Helsinki, the guidelines of Good Clinical Practice (GCP) issued by ICH, and Swiss Law and Swiss regulatory authority’s requirements. The CEC and regulatory authorities will receive Annual Safety and interim Reports and be informed about study stop/end in agreement with local requirements.

## Declaration of interest

The principle investigator as well as all other involved third parties declares no conflict of interest. All involved parties received no other financial support than stated within this document.

## Patient Information and Informed Consent

The investigators will explain to each participant the nature of the study, its purpose, the procedures involved, the expected duration, the potential risks and benefits and any discomfort it may entail. Each participant will be informed that the participation in the study is voluntary and that he/she may withdraw from the study at any time and that withdrawal of consent will not affect his/her subsequent medical assistance and treatment.

The participant must be informed that his/her medical records may be examined by authorised individuals other than their treating physician.

All participants for the study will be provided a participant information sheet and a consent form describing the study and providing sufficient information for participant to make an informed decision about their participation in the study. No emergency operations are included in this study. All participants are given at least 72h of time to decide whether to participate or not in the study.

The patient information sheet and the consent form will be submitted to the CEC to be reviewed and approved. The formal consent of a participant, using the approved consent form, must be obtained before the participant is submitted to any study procedure.

The participant should read and consider the statement before signing and dating the informed consent form, and should be given a copy of the signed document. The consent form must also be signed and dated by the investigator (or his designee) and it will be retained as part of the study records.

## Participant privacy and confidentiality

The investigator affirms and upholds the principle of the participant's right to privacy and that they shall comply with applicable privacy laws. Especially, anonymity of the participants shall be guaranteed when presenting the data at scientific meetings or publishing them in scientific journals.

Individual subject medical information obtained as a result of this study is considered confidential and disclosure to third parties is prohibited. Subject confidentiality will be further ensured by utilising subject identification code numbers to correspond to treatment data in the computer files.

For data verification purposes, authorised representatives of the Sponsor (-Investigator), or the ethics committee may require direct access to parts of the medical records relevant to the study, including participants’ medical history.

The study case reporting form is assigned with a study number. From this number no conclusions to the identity (name, birth date) of the participant may be drawn. The key file to decrypt the study number is stored with the sponsor-investigator separately form the CRF form.

## Early termination of the study

The Sponsor-Investigator may terminate the study prematurely according to certain circumstances, for example:

- ethical concerns,
- insufficient participant recruitment,
- when the safety of the participants is doubtful or at risk, respectively,
- alterations in accepted clinical practice that make the continuation of a clinical trial unwise,
- early evidence of benefit or harm of the experimental intervention

## Protocol amendments

Substantial amendments are only implemented after approval of the CEC and CA respectively.

Under emergency circumstances, deviations from the protocol to protect the rights, safety and well-being of human subjects may proceed without prior approval of the sponsor and the CEC/CA. Such deviations shall be documented and reported to the sponsor and the CEC/CA as soon as possible.

All non-substantial amendments are communicated to the CA as soon as possible if applicable and to the CEC within the Annual Safety Report.

# Background and Rationale

## Background and Rationale

Surgical procedures for inguinal hernia repair

The primary and only definitive treatment of inguinal hernia is surgery. The surgical techniques have changed in the past decades. For a long period, open techniques were the gold standard, initially with reconstruction of the wall layer (e.g. Bassini, Shouldice) later with tension-free mesh implantation (Lichtenstein procedure). In the last decade different minimal invasive surgery (MIS) techniques such as laparoscopic totally extra-peritoneal (TEP) and transabdominal pre-peritoneal (TAPP) inguinal hernia repair showed good results considering low postoperative pain and infrequent surgical site infections. Whereas open tension-free methods show a clear benefit over open meshless techniques with wall reconstruction, the superiority of MIS over the Lichtenstein mesh implantation technique is still controversial. Surgery for inguinal hernia belongs to one of the most often performed surgeries in developed countries and clear evidence is eminent. A recent systemic review comparing surgical methods by Aiolfi concluded, that “the surgical management of inguinal hernia is evolving and the effect of the adoption of innovative minimally invasive techniques should be further investigated”. Robotic transabdominal pre-peritoneal (rTAPP) inguinal hernia repair represents such a technique. Thanks to the excellent stereotactic vision and the gentle preparation with additional articulation in perfect anatomic layers with potential sparing of nerve injury, less tissue trauma and less blood loss could lead to superior postoperative outcomes.

Current understanding of postoperative outcome in inguinal hernia repair

The rate of hernia recurrence and postoperative pain are of great importance. A recent meta-analysis by Lyu Y. comparing endoscopic and Lichtenstein repair showed no differences among TAPP, TEP and Lichtenstein in terms of hernia recurrence and chronic pain. Other studies, tough, have found pain to be less after endoscopic hernia repair compared to open repair. However, still, approximately 10-15% report pain one year after surgery, depending on the applied method.2

A recurrent hernia itself often requires another surgery and is associated with a high incidence of chronic pain as published in a single-blinded randomized trial by Lydeking. Köckerling even showed in an analysis of 16,206 patients that postoperative complications continuously increased after multiple recurrences (first recurrence: 3.97% vs second recurrence: 5.75% vs ≥ third recurrence 8.65%). Similarly, the rate of pain requiring treatment rose highly significantly with an increasing number of recurrences (first recurrence: 5.21% vs second recurrence: 6.70% vs ≥ third recurrence 10.86. Other early onset complications pose surgical site infections (SSI), wound seromas and hematomas. Many different factors may influence the appearance of complications and are subject to a large number of clinical investigations. Besides primary surgical approach (e.g. open or minimally invasive), the fixation technique of the mesh can influence the complication rate. In general MIS can minimize the risk of local wound complications such as hematoma and seroma in current literature. The earlier mentioned meta-analysis by Lyu Y showed no differences in regard of hematoma, seroma and hospital stay though. Lichtenstein had a shorter operation time than TAPP + TEP but more wound infections than TEP; TAPP and TEP required fewer return-to-work days.

Whether robotic surgery may generate even better results is currently a matter of debate. Besides local wound problems, (chronic) pain is of eminent importance for patients undergoing groin hernia repair. Postoperative pain can influence the quality of life (QOL) of the individual patient substantially. Chronic postoperative pain can be caused by intraoperatively nerve damage or impingement. Besides direct intraoperative damage, postoperative shrinking of mesh material, mesh material itself, mechanical irritation or mesh fixation technique influence the appearance of chronic discomfort as well.16 Consequently, surgeons began to resect the inguinal nerves in the open approach; however this modification of the procedure could not achieve satisfactory results in the long term.

F. Köckerling states severe postoperative pain as an important risk factor for the onset of chronic inguinal pain and suggests early surgical intervention should be considered for patients with severe postoperative pain > 3 days.

Although our literature research reveals the importance of acute postoperative pain for the development of chronic pain, we will not be able to show any direct difference in the occurrence of chronic pain with the proposed trial. Whereas chronic pain remains a key topic, it still decreased over postoperative time and a much larger and not realistic trial is needed to show a direct benefit of robotic surgery.

## Investigational Product (treatment) and Indication .

n.a. since the da Vinci Xi robot is certified and approved by American and European authorities for the proposed surgery.

## Preclinical Evidence

n.a. since rTAPP is already widely used around the world.

## Clinical Evidence to Date

There is enough clinical expertise to conclude that rTAPP is safe. It is widely in use independent of this study. Also we already offer rTAPP to patients if indicated. There are no clinical studies to our knowledge that investigate our endpoints for rTAPP. Clinical evidence therefore lacks behind clinical practice.

## Rationale for the dosage, route, regimen

n.a.

## Explanation for choice of comparator (or placebo)

Endoscopic technique for the treatment of inguinal hernias are nowadays regarded as equivalent or even superior compared to open techniques. In the last decade different minimal invasive surgery (MIS) techniques such as laparoscopic totally extra-peritoneal (TEP) and transabdominal pre-peritoneal (TAPP) inguinal hernia repair showed good results considering low postoperative pain and infrequent surgical site infections. TEP was chosen as a comparator because this technique is the most established in our department and involved surgeons are experienced in TEP. The outcome of TAPP and TEP is comparable. Intraoperative and postoperative complications as well as the reoperation rate are not different in TAPP versus TEP. In the guidelines of the European Association for Endoscopic Surgery TAPP and TEP are both equally recommended for the treatment of inguinal hernias.

## Risks / Benefits

The participation in this clinical trial is based on oral and written informed consent and is voluntary. The surgical techniques are not modified in case of participation to this trial. TEP (control group) and rTAPP (intervention group) have both been performed and published and are considered to be safe. Both techniques have already been performed numerous times at our institution. The primary and only definitive treatment of inguinal hernia is surgery. There are no specific benefits and risks related to this trial. Risks related to the surgery are independent to this trial and patients are informed about the procedure and these risks independently of the trial.

## Justification of choice of study population

In the trial patients undergoing routine procedures in minimal invasive inguinal hernia repair are included. No vulnerable patients or patients with an inability to informed consent are included. No emergency operations are included.

# STUDY OBJECTIVES

## Overall Objective

Endoscopic technique for the treatment of inguinal hernias are nowadays regarded as equivalent or even superior compared to open techniques. Nevertheless, techniques such TEP and TAPP are technically demanding. The learning curve for both of them is quite long. It takes more than 60 procedure to achieve a reduction in procedure time and complications.

Moreover, pain after inguinal hernia repair is a major issue as it may compromise the quality of life of an otherwise generally young, working population. Although pain is estimated to be less after endoscopic hernia repair compared to open repair, still, 15% report pain one year after surgery. Robotic TAPP has emerged in recent years and may allow more meticulous preparation and thus better preserve sensory nerves. Fixation of the very superficial sutures and better closure of the peritoneum became feasible with rTAPP. Thus, the objective of the current randomized controlled trial is to investigate whether rTAPP is associated with a decreased pain level shortly after surgery compared to conventional TEP. Acute pain is a surrogate for later chronic pain and for postoperative recovery. Specific further aims are the comparison of procedural costs and overall healthcare costs in the two techniques. So far, no randomized clinical trial comparing rTAPP with conventional endoscopic techniques have has been published.

The aim of this study is to evaluate whether a robot-assisted inguinal hernia repair can reduce the postoperative pain compared to the conventional laparoscopic approach.

## Primary Objective

With this randomized and blinded trial we compare rTAPP to conventional TEP. The primary objective is to study a possible reduction in postoperative pain. Pain at coughing 24 hours after surgery measured on a numeric rating scale (NRS 0-10) while coughing. A 20% reduction in mean pain at coughing 24 hours after surgery is considered clinically significant. On this assumption the sample size calculation is based on.

## Secondary Objectives

Secondary objectives of this trial are to study pain additional times postoperative (2h, day 7, day 30, 6 and 12 months) with help of the NRS and the sf-IPQ. Furthermore, patients are examined clinically and if in doubt by ultrasound for the rate of recurrence 6 and 12 months after surgery. To get a larger picture of the patient’s course quality of life will be investigated with different scores (EQ-5D-5L, SF-6D, ICECAP-O, CCS, SF-12).

Complications are measured with the Comprehensive Complication Index (CCI).

One argument often discussed in the context of robotic surgery is the superior ergonomics for the surgeon. We will study this objective by questioning the surgeon (NASA TLX, specific questionnaire) before and after surgery.

Another hot topic in robotic surgery are the costs. In this trial we will address the cost per surgery per patient as well as the costs for the institution and economic impact (sick leave). This will be estimated by the days until resumption of work or days until resumption of activities of daily life. For sick leave costs the days will be multiplied by average daily costs of sick leave in Switzerland according to the Swiss National Accident Insurance Fund (SUVA). The type of labor including the relative activity level (sedentary work, light work, medium work, heavy work, very heavy work, retired/unemployed) according to US Code of Federal Regulations § 404.1567 Physical exertion requirements) is included in the evaluation process.

## Safety Objectives

No safety objectives are investigated in this study. However, intra- and perioperative morbidity in both groups will be recorded.

# STUDY OUTCOMES

Describe the primary, secondary, and other outcomes, including the specific measurement variable (e.g., systolic blood pressure or description of surrogate marker for non-measurable variables), analysis metric (e.g., change from baseline, final value, time to event), time point for each outcome etc. Explanation of the clinical relevance of chosen efficacy and harm outcomes is strongly recommended.

## Primary Outcome

Pain at coughing 24 hours after surgery measured on a numeric rating scale (NRS 0-10) while coughing.

## Secondary Outcomes

The following scores are used to objectify secondary outcomes:

- NRS score 2 hours, 24 hours (at rest), 7 days and 30 days, 6 and 12 months after surgery at rest and while coughing
- Inguinal pain questionnaire (sf-IPQ) 24 hours, 7 days and 30 days, 6 and 12 months after surgery
- EQ-5D-5L 24 hours, 7 days and 30 days, 6 and 12 months after surgery
- SF-6D 24 hours, 7 days and 30 days, 6 and 12 months after surgery
- ICECAP-O 24 hours, 7 days and 30 days, 6 and 12 months after surgery
- Intraoperative complications
- Amount of perioperative pain medication
- Procedure time
- Time in the OR block
- For day surgery: hours in outpatient clinic until discharge
- For hospitalized patients: postoperative stay in days
- Prescribed and actually taken pain medication postoperative 24 hours, 7 days and 30 days, 6 and 12 months after surgery
- Postoperative morbidity classified according to the Dindo-Clavien classification and scored according to the Comprehensive complication index (CCI) up to 30 days after surgery.
- Recurrence rate 6 and 12 months postoperative. Recurrences are excluded clinically and by sonography by the operating surgeon (face-to-face visit).
- Quality of life (SF-12) 30 days, 6 months, and 12 months postoperative.
- Carolinas Comfort Scale (CCS) 30 days, 6 months, and 12 months postoperative.
- Ergonomics for the surgeon measured by NASA TLX and specific questionnaire.
- Experience of the surgeons in rTAPP and/or TEP (performed surgeries in the last 12 months)
- Costs for surgery per patient according to the accounting department (costs for care, operating room, anesthesia, consumables)
- Sick leave (days until resumption of work or days until resumption of activities of daily life, and estimated sick leave by patient).
- Costs for sick leave (days multiplied by average daily costs of sick leave in Switzerland according to the Swiss National Accident Insurance Fund (SUVA)).
- Type of labor including the relative activity level (sedentary work, light work, medium work, heavy work, very heavy work, retired/unemployed) according to US Code of Federal Regulations § 404.1567 Physical exertion requirements.

## Safety Outcomes

No safety outcomes are assessed in this study. Intra and postoperative morbidity will be assessed.

# STUDY DESIGN

## General study design and justification of design

The aim of this study is to evaluate whether rTAPP can reduce postoperative pain compared to TEP.

We decided for a prospective, randomized and blinded clinical trial because of its superiority compared to other study designs regarding minimizing biases. We will be able to include the necessary count of patients based on our power calculation and by checking the performed surgeries over the last years. To our knowledge no such study has been performed to investigate our primary objective.

All patients in need of an elective minimal invasive hernia surgery and who meet the inclusion criteria and exclusion criteria do not apply will be included after orally and writing informed, at least 72 hours before surgery and willing of participating (by signing the informed consent).

Patients are assigned by internet-based randomization [1. Ltd, S.E., Simple randomisation service. https://http://www.sealedenvelope.com/simple-randomiser(v1, 2015] to the intervention or control group. There will be a 1:1 randomization. Randomization will take place after outpatient consultation by an independent case manager. Neither the involved surgeon, nor the patient are involved in the randomization.

## Methods of minimising bias

### Randomisation

- Patient are allocated to rTAPP or TEP by randomization. Randomization is performed by block randomization and stratified by BMI (<30kg/m2; ≥30kg/m2) and for unilateral or bilateral hernia. Despite a 1:1 randomisation to rTAPP or TEP, randomisation is stratified for uni- and bilateral hernias to ensure equal distribution in both groups. It is performed using an internet-based stratified block randomization software (www.sealedenvelope.com).
- Ideally, the randomization would be performed just before incision. Yet, due to limited slots for the da Vinci robot for hernia surgery it is mandatory to perform the randomization beforehand. According to the randomization patients will be allocated to rTAPP or TEP slot.
- Randomization will be performed by a case manager that is not involved in the treatment of the patient.
- The randomization does not have an influence on the allocation to the surgeon. The surgeon that has counseled the patient prior to study inclusion and randomiziation will perform the procedure regardless of the result of the randomization.
- Patients and assessors of study variables are blinded for the randomization.

### Blinding procedures

- For rTAPP three incision are made in horizontal line at the level of the umbilicus. For TEP three incisions are made in a vertical line between the symphysis and the umbilicus. All patients will receive the same standardized opaque dressing covering all potential incisions of rTAPP as well as TEP. This allows blinding of the patients and the assessors of study variables. The study nurse that assesses the primary endpoint (NRS 24h) as well as secondary outcomes is blinded for the allocation to rTAPP or TEP. Patients will be unblinded 7 days after surgery. This is done at the end of the 7 days follow up by phone. We decide to unblind patients after 7 days, because the primary endpoint is recorded by that time and patients will be able to determine the performed surgery by the scars (Steristrips begin to fall of).
- Violation of blinding will be noted in the case reporting form with the reason for premature unblinding.

### Other methods of minimising bias

Validate questionnaires and scores will be used for assessing the quality of life and the pain of the patients (see CRF).

## Unblinding Procedures (Code break)

The used randomisation procedure and respective allocation will be documented by the case manager. The randomisation protocol is recorded within the randomisation software and includes the patient study identification number only. Patients will be unblinded 7 days after surgery. They will be unblinded if there is medical or surgical complication requiring unblinding or at the patient’s request at any time. Violation of blinding will be noted in the case reporting form with the reason for premature unblinding.

# STUDY POPULATION

## Eligibility criteria

The study population will be patients with an indication for an elective minimal invasive repair of inguinal hernia.

Participants fulfilling all of the following inclusion criteria are eligible for the study:

- Patients older than 18 years of age and able to understand and give their informed consent for the study.
- Primary unilateral or bilateral hernia

The presence of any one of the following exclusion criteria will lead to exclusion of the participant:

- Recurrent hernia
- with previous open abdominal surgery at or below the umbilicus
- need of an open inguinal hernia repair (patient’s preference, unable to undergo general anesthesia, unable to tolerate pneumoperitoneum)
- liver disease defined by the presence of ascites
- end-stage renal disease requiring dialysis
- unable to give informed consent
- need of an emergency surgery
- pregnancy

## Recruitment and screening

We will screen all patients undergoing planned inguinal hernia surgeries at our department for inclusion criteria. To ensure a high inclusion rate only investigators inform patients about this trial. Once patients give informed consent and exclusion criteria to not apply, patients will be randomized. No payment or compensation to the study participants is provided.

## Assignment to study groups

Patient are allocated to rTAPP or TEP by randomization. Randomization is performed in a 1:1 fashion to rTAPP or TEP. It is performed using an internet-based block randomization software (www.sealedenvelope.com). Randomization will be performed by a case manager that is not involved in the treatment of the patient. The randomization does not have an influence on the allocation to the surgeon.

## Criteria for withdrawal / discontinuation of participants

Patients are withdrawn from the study at any time if informed consent is withdrawn or if the study is prematurely stopped for safety concerns. In case of individual withdrawal of informed consent or loss to follow-up, the patients will be replaced.

# STUDY INTERVENTION

## Identity of Investigational Products

### Experimental Intervention

Due to Swiss regulations, surgery for patients with a unilateral hernia is carried out outpatient whereas patients with bilateral hernias are hospitalized for at least one night. All patients receive intravenous antibiotic prophylaxis with cefazolin 1g 30 minutes before incision. All patients receive standardized analgesics treatment after surgery: celecoxib 200mg p.o. b.i.d . for 5 days and metamizol 500mg p.o. q.i.d. for 7 days.

rTAPP: Patients are under general anesthesia and receive 2g cefazolin antibiotic prophylaxis prior to incision. The Xi da Vinci patient cart is placed to either side of the operating table with integrated table motion. The first 8-mm robotic port with an Airseal insufflator is placed at the umbilicus after insufflation of carbon dioxide by a Verres needle. After a brief laparoscopy another two 8-mm robotic ports are placed on either side of the umbilicus between 6–8 cm in distance to each other. After placing the patient in a slight Trendelenburg position, we dock the Xi robot. The right port holds the monopolar scissors, the left port the fenestrated bipolar grasper. In the center port, we place a 30° camera. The peritoneum is incised 4-5 cm above the internal ring from the median umbilical ligament in direction of the anterior superior iliac spine and gradually detached from the transversalis fascia. While medially we enter the space of Retzius and expose the Cooper ligament, laterally we dissect to the psoas muscle. To reduce an indirect hernia the sac is separated from the vas deferens and the vessels. A direct hernia will be grasped reduced. The peritoneum is parietalized for another 5 cm in cephalad direction at least to create space for the mesh. We always check for a femoral hernia. We place a mesh of at least 15x12cm to cover the entire myopectineal orifice including direct, indirect and femoral spaces. The mesh is sutured to the Cooper ligament and in case of a larger medial hernia additionally to the ventral abdominal wall with an absorbable Vicryl. The triangle of pain is spared. The peritoneal flap is attached with a 3-0 absorbable V-Lock running suture. The ports will be removed and gas desufflated. The Skin is closed with a resorbable intracutaneous suture.

### Control Intervention (standard/routine/comparator treatment)

Due to Swiss regulations, surgery for patients with a unilateral hernia is carried out outpatient whereas patients with bilateral hernias are hospitalized for at least one night. All patients receive intravenous antibiotic prophylaxis with cefazolin 1g 30 minutes before incision. All patients receive standardized analgesics treatment after surgery: celecoxib 200mg p.o. b.i.d . for 5 days and metamizol 500mg p.o. q.i.d. for 7 days.

TEP: Patients are under general anesthesia and receive 2g cefazolin antibiotic prophylaxis prior to incision. Incision at the umbilicus to gain entry below the rectal muscle space is carried out. After a dissecting balloon is introduced towards the symphysis to create preperitoneal space under camera vision a 12 mm port is placed. Another two 5 mm ports are installed along the median line below the umbilicus. The patient is placed in a slight Trendelenburg position and tilt towards the surgeon. The Cooper ligament is visualized and a femoral hernia is looked for. A direct hernia will be grasped reduced and an indirect hernia sac is separated from the vas deferens and the vessels for reduction. Further preparation and placement of the mesh is performed accordingly to the steps described in the robotic part. The mesh is fixed with resorbable tackers or glued. The ports will be removed and gas desufflated. The fascia at the umbilicus is closed with Vicryl and the skin with a resorbable intracutaneous suture.

### Packaging, Labelling and Supply (re-supply)

n.a.

### Storage Conditions

n.a.

## Administration of experimental and control interventions

### Experimental Intervention

See Chapter 8.1.1.

### Control Intervention

See Chapter 8.1.2.

## Dose modifications

Not applicable for this trial.

## Compliance with study intervention

In this study, patients with a primary unilateral hernia and no risk factors are treated outpatients according to current AVOS regulations [https://www.gesundheitsversorgung.bs.ch/gesundheitsfachpersonen/spitalversorgung/spitalfinanzierung/avos.html]. All other participants in this trial (active/control group) will stay in hospital for at least 24 hours (not affected by the study participation). During this period data will be collected through medical or nursing staff. After hospital discharge, the monitoring will be done independently by the participant be filling questionnaires and check up by phone or face-to-face according to the study schedule.

To ensure a high follow up rate patients are personally addressed by mail and phone for consultations. If not reached, we try calling two more times at another time and day. If a patient is not to be located after that, we get in contact with the family doctor to get more information.

No compliance is defined as when despite all above mentioned measures more than 50% of the questionnaires in the schedule plan within 2 months after the last follow up are not returned. The non-compliant participant will be withdrawn from the study.

## Data Collection and Follow-up for withdrawn participants

The questionnaire given to the patient should be collected (through letter mail) within 2 months to the last follow-up according to the study schedule.

## Trial specific preventive measures

No restrictions on medications or treatments are supposed in this study. All patient satisfying the inclusion criteria are potential participants.

## Concomitant Interventions (treatments)

No additional surgical intervention during the observational period should be undertaken.

## Study Drug Accountability

The da Vinci Xi surgical robot is already widely used to perform hernia surgeries in Switzerland and worldwide. No additional approval specific to this study will be needed for medical devices. The da Vinci Xi has already been used for the exact same procedure (rTAPP) before and independently of this trial at our institution.

## Return or Destruction of Study Drug

The da Vinci Xi is already used at our institution and standard procedures for destruction (single-use material) or processing for re-use (multi-use material) are already put in place.

# STUDY ASSESSMENTS

## Study flow chart(s) / table of study procedures and assessments

|  | Baseline | Day 0, 2h postop | 24h postop | 7d postop | 30d postop | 6 months | 12 months |
| --- | --- | --- | --- | --- | --- | --- | --- |
| Assessment | Face-to-face | Face-to-face | Telephone | Telephone | Telephone | Face-to-face | Face-to-face |
| NRS (primary endpoint) | + |  | + |  |  |  |  |
| NRS |  | + |  | + | + | + | + |
| sf-IPQ | + |  | + | + | + | + | + |
| EQ-5D-5L | + |  | + | + | + | + | + |
| ICECAP-O | + |  | + | + | + | + | + |
| morbidity |  |  |  |  | + |  |  |
| Intraoperative complications |  | + |  |  |  |  |  |
| Perioperative pain medication |  | + |  |  |  |  |  |
| OR and procedure time |  | + |  |  |  |  |  |
| Hospital stay |  | + |  |  |  |  |  |
| Pain medication (prescribed and intake) |  | + | + | + | + | + | + |
| Recurrence |  |  |  |  |  | + | + |
| SF-12 | + |  |  |  | + | + | + |
| CCS | + |  |  |  | + | + | + |
| SF-6D | + |  | + | + | + | + | + |
| NASA TLX | + | + |  |  |  |  |  |
| Specific questionnaire | + | + |  |  |  |  |  |
| Costs for surgery |  |  |  |  | + |  |  |
| Sick leave |  |  |  |  | + |  |  |
| Costs for sick leave |  |  |  |  | + |  |  |

## Assessments of outcomes

### Assessment of primary outcome

The primary outcome of this study is pain at coughing 24 hours after surgery measured on a numeric rating scale (NRS 0-10) while coughing. This will be assessed through NRS at 24 hours after the intervention (see CRF).

### Assessment of secondary outcomes

The following scores are secondary endpoints:

- NRS score 2 hours, 7 days and 30 days, 6 and 12 months after surgery.
- Inguinal pain questionnaire (sf-IPQ) 24 hours, 7 days and 30 days, 6 and 12 months after surgery
- EQ-5D-5L 24 hours, 7 days and 30 days, 6 and 12 months after surgery
- SF-6D 24 hours, 7 days and 30 days, 6 and 12 months after surgery
- ICECAP-O 24 hours, 7 days and 30 days, 6 and 12 months after surgery
- Intraoperative complications
- Amount of perioperative pain medication
- Procedure time
- Time in the OR block
- For day surgery hours in outpatient clinic until discharge
- For hospitalized patients postoperative stay in days
- Prescribed and actually taken pain medication postoperative 24 hours, 7 days and 30 days, 6 and 12 months after surgery
- Postoperative morbidity classified according to the Dindo-Clavien classification and scored according to the Comprehensive complication index (CCI) up to 30 days after surgery.
- Recurrence rate 6 and 12 months postoperative. Recurrences are excluded clinically and by sonography by the operating surgeon (face-to-face visit).
- Quality of life (SF-12) 30 days, 6 months, and 12 months postoperative.
- Carolinas Comfort Scale (CCS) 30 days, 6 months, and 12 months postoperative.
- Ergonomics for the surgeon measured by NASA TLX and specific questionnaire.
- Experience of the surgeons in rTAPP and/or TEP (performed surgeries in the last 12 months)
- Costs for surgery per patient according to the accounting department (costs for care, operating room, anesthesia, consumables)
- Sick leave (days until resumption of work or days until resumption of activities of daily life, and estimated sick leave by patient).
- Costs for sick leave (days multiplied by average daily costs of sick leave in Switzerland according to the Swiss National Accident Insurance Fund (SUVA)).
- Type of labor including the relative activity level (sedentary work, light work, medium work, heavy work, very heavy work, retired/unemployed) according to US Code of Federal Regulations § 404.1567 Physical exertion requirements.

### Assessment of other outcomes of interest

We are investigating patients outcomes and the surgeon’s experience. All measured endpoints are listed in 9.2.2.

### Assessment of safety outcomes

The study does not investigate safety outcomes. However, intra- and postoperative outcomes will be recorded in both groups.

### Assessments in participants who prematurely stop the study

These patients are followed according to standard routine. Patients after inguinal hernia repair are followed-up by their family doctor and are referred to us with a low threshold in case of any uncertainty or unforeseen event.

## Procedures at each visit

### Screening visit

At the screening visit the medical history and a physical examination will be done. The indication for surgery is made and patients are informed about it. By inclusion criteria, the patient will be orally and in writing informed about the study. Baseline data will be collected according to the chart columne “baseline” in Chapter 9.1. Paperwork for “Informed consent” will be explained and given to the patient. If patients return the “informed consent” by mail, they will be randomized by an independent case manager. Patients will be handed out an explanation of the NRS and it is orally explained in addition.

### Admission time

Patients will enter the hospital at the day of the surgery. The responsible study doctor or an experienced colleague from the surgical ambulatory will visit the patient to make sure, no changes in clinical presentation of the hernia or the general health condition occurred between the screening visit/outpatient clinic and the day of admission. Further and last questions before surgery are investigated and answered.

### In the operating room, before incision

Since patients are randomized before hospital admission, all preoperative procedures are carried out in a standardized way. Study parameters are documented as above-mentioned during the intervention.

Surgeon assessment as mentioned in Table 9.1.

### Surgical intervention

The surgeon will proceed to surgical intervention. All vital parameters (blood pressure, pulse, temperature, blood oxygenation, amount of pain killers) are documented by the anaesthesia care team during the intervention independently of this study. However, we will take these parameters into account for our analysis.

Direct postoperatively surgeon assessment as mentioned in Table 9.1.

### Early postoperative (up to 24h postop)

The NRS as primary endpoint is assessed after 24 hours. Independently if patients will leave the hospital on the day of the surgery (unilateral hernia) or if they will stay for one night (bilateral hernia/unilateral complicated/unilateral and comorbidities), the study nurse will assess them 24h postoperatively by phone. The 2h postoprative NRS is also conducted by the study nurse. Additionally, the last time point of the surgeons assessment by above-mentioned scores is conducted at the 24 hours time point.

### Follow-up (24h – 12 months postop)

For the outcome assessment after the 24 hours time point patients will be contacted by phone from the study nurse (day 7 and day 30) or personally in our outpatient clinic (6 months and 12 months). The questionnaires will reach the patients by mail, a separate return envelope will be added to avoid additional costs for the participant. The assessed variables can be found on table 9.1. in this manuscript.

# SAFETY

## Medical Device Category A studies

### Definition and Assessment of safety related events

Findings in the trial that may affect the safety of study participants and which require preventive or corrective measures intended to protect the health and safety of study participants.

An **Adverse Event (AE)** is any untoward medical occurrence in a patient or a clinical investigation participant administered a pharmaceutical product and which does not necessarily have a causal relationship with the study procedure. An AE can therefore be any unfavourable and unintended sign (including an abnormal laboratory finding), symptom, or disease temporally associated with the use of a medicinal (investigational) product, whether or not related to the medicinal (investigational) product. [ICH E6 1.2]

A **Serious Adverse Event (SAE)** is classified as any untoward medical occurrence that:

- results in death,
- is life-threatening,
- requires in-patient hospitalization or prolongation of existing hospitalisation,
- results in persistent or significant disability/incapacity, or
- is a congenital anomaly/birth defect.

In addition, important medical events that may not be immediately life-threatening or result in death, or require hospitalisation, but may jeopardise the patient or may require intervention to prevent one of the other outcomes listed above should also usually be considered serious. [ICH E2A]

SAEs should be followed until resolution or stabilisation. Participants with ongoing SAEs at study termination (including safety visit) will be further followed up until recovery or until stabilisation of the disease after termination.

Assessment of Causality

Both Investigator and Sponsor-investigator make a causality assessment of the event to the study drug, based on the criteria listed in the ICH E2A guidelines:

| Relationship | Description |
| --- | --- |
| Definitely | Temporal relationship  Improvement after dechallenge*  Recurrence after rechallenge  (or other proof of drug cause) |
| Probably | Temporal relationship  Improvement after dechallenge  No other cause evident |
| Possibly | Temporal relationship  Other cause possible |
| Unlikely | Any assessable reaction that does not fulfil the above conditions |
| Not related | Causal relationship can be ruled out |
| *Improvement after dechallenge only taken into consideration, if applicable to reaction | |

Unexpected Adverse Drug Reaction

An “unexpected” adverse drug reaction is an adverse reaction, the nature or severity of which is not consistent with the applicable product information (e.g. Investigator’s Brochure for drugs that are not yet approved and Product Information for approved drugs, respectively). [ICH E2A]

Suspected Unexpected Serious Adverse Reactions (SUSARs)

The Sponsor-Investigator evaluates any SAE that has been reported regarding seriousness, causality and expectedness. If the event is related to the investigational product and is both serious and unexpected, it is classified as a SUSAR.

Assessment of Severity

The grades for severity described in the “Common Terminology Criteria for Adverse Events CTCAE Version 5.0 (27.11.2017 U.S. Department of health and human services) are applied.

### Reporting of Safety related events

Reporting to Sponsor-Investigator:

Health hazard that require measures are reported to the Sponsor-Investigator within 24 hours upon becoming aware of the event.

Reporting to Authorities:

In Category A studies it is the Investigator’s responsibility to report to the local Ethics Committee. Health hazards that require direct measures are reported to the ethics committee in 24 hours. Excluded are effects unrelated to the operation method (e.g. allergic reaction on administered drugs). The decision if adverse events are related to the operation method and consequently need to be reported is the measure of the principle investigator.

# STATISTICAL METHODS

## Hypothesis

The hypothesis is a 20% reduction of the NRS while coughing 24 hours after surgery. Short-term postoperative pain is a surrogate marker for the duration of sick leave and of prolonged postoperative pain.

## Determination of Sample Size

Based on previous studies investigating pain at cough 24 hours after surgery we estimate the NRS level to be about 4.37 ± 1.66. This estimate takes into consideration two previous studies. The first study is a double-blinded randomized controlled trial by A. Mughal analyzed 62 patients that underwent TEP with and without peripheral nerve block. The second study is a retrospective study analysis of 583 patients that underwent TEP by Chih-Chin comparing the mean NRS after TEP with glue versus tacker fixation of the mesh. For our calculations, we used the mean scores of these two studies including the intervention as well as the comparison group. The used numbers are therefore independent from the type of mesh fixation. In a systematic review seeking the answer for the minimum clinically important difference (MCID) in acute pain measured by VAS or NRS authors came to the conclusion MCID is context-specific. By looking at the included studies a relative pain reduction 10-30% was clinically significant. In the sample size calculation, a 20% reduction of the mean number was used. Short-term postoperative pain is a surrogate marker for recovery (sick leave) and of prolonged postoperative pain.

An NRS of 4.37 ± 1.66 24 hours after surgery at coughing in the control group is assumed. The study hypothesis is a 20% reduction of pain at coughing on postoperative day 1. Given a level of significance of 0.05 and a power of 0.90 a sample size of 76 patient per group are needed. A drop out rate of 20% is estimated. Therefore we plan to include a total of 182 patients.

## Statistical criteria of termination of trial

No interim analysis is planned for this trial. Analysis will be performed after inclusion and follow-up of the last patient. The primary investigator performs the analysis with help of the co-investigators.

## Planned Analyses

Descriptive statistics (means, medians, quartiles, ranges, standard deviations) will be performed to assess the demographics of the patient population and the questionnaires.

Primary and secondary outcomes in the per-protocol population will be analyzed. Differences between continuous variables will be assessed using the t-test. For multi-group comparisons differences will be assessed using the Kruskal-Wallis or the one-way ANOVA test depending on normality of distribution of comparison groups. Chi square test or Fisher’s exact test will be used for comparisons regarding the categorial endpoints. The normal distribution will be controlled by Kolmogorov-Smirnov test. After cross-tabulating results they will be also filtered so that data may be further modeled and thereby a subgroup analysis may be performed according to the descriptive characteristics of the study population. To show trends a simple linear regression will be analyzed.

The level of significance will be set 0.05. The primary investigator will perform the analysis in collaboration with a statistician.

### Datasets to be analysed, analysis populations

All subjects participating the study (without withdraw) will be analysed.

### Primary Analysis

Pain at coughing 24 hours after surgery measured on a numeric rating scale (NRS 0-10) while coughing. A 20% reduction in mean pain at coughing 24 hours after surgery is considered clinically significant.

### Secondary Analyses

Secondary analyses are measured with the NRS and different quality of life scores (sf-IPQ, EQ-5D-5L, SF-6D, ICECAP-O, CCS, SF-12) at different times (see secondary objectives in section 4.3) for patients.

Complications are measured with the Comprehensive Complication Index (CCI).

Surgeon’s ergonomics, stress and satisfaction is measured with the NASA TLX and specific questions

Costs will be addressed as cost per surgery per patient (billing), costs for the institution (internal costs), and as economic impact (sick leave). This will be estimated by the days until resumption of work or days until resumption of activities of daily life. For sick leave costs the days will be multiplied by average daily costs of sick leave in Switzerland according to the Swiss National Accident Insurance Fund (SUVA).

### Interim analyses

n.a.

### Safety analysis

n.a.

### Deviation(s) from the original statistical plan

Describe how any deviation(s) from the planned analyses will be justified and reported.

## Handling of missing data and drop-outs

Missing data will be reported. Drop-out of patients will be replaced.

# QUALITY ASSURANCE AND CONTROL

## Data handling and record keeping / archiving

Data is documented in a study CRF and archived in the responsibility of the primary investigator. Data is stored with the sponsor-investigator and kept in Clarunis, Basel. The CRF forms are coded. The key file, which allows identification of the participants, are stored separately. According to the regulation study forms are stored for 10 years after termination of the study. Afterwards they are destroyed.

### Case Report Forms

Paper Case Report Forms (CRF) are used. Each participant receives a study number. No conclusion on the participants’ identity may be drawn out of the study number, e.g. initials, name or birth date are not included in the study number. The key file with the allocation of the study numbers to the individual participants is stored with the sponsor-investigator separately from the CRFs. The key file is only accessible to the sponsor-investigator and to the relevant authorities. At the end of the study, anonymized data of the CRF are put in a REDcap database by the investigator. Data entry is controlled by the co-investigators.

### Specification of source documents

Source data are considered the patients’ electronic medical history, reports from the outpatient clinic, operating room reports, the paper case reporting form, electronic information about study allocation (randomization) and the signed informed consent. The source documents in paper form are archived by the primary investigator in the study center; electronic data are stored in the electronic clinical information system.

### Record keeping / archiving

All study documents must be archived for a minimum of 10 years after study termination or premature termination of the clinical trial.

The above-mentioned documents are archived for 10 years after termination of the study.

## Data management

All study variables are documented in the paper case reporting form. After recruiting the data are entered in a REDCap database. Responsible for the data management is the primary investigator.

### Data Management System

REDCap database is used to manage data. The system is hosted within Clarunis at the University Hospital Basel. Responsible for the data management is the primary investigator Clarunis. REDCap is already installed and used for several other studies. We run on the server before trial start.

### Data security, access and back-up

The principal investigator, the co-investigators and the study nurse have access to the coded REDCap database. The principal investigator may grant limited access to other persons involved in data analysis (statistician e.g.). Once data analysis is performed all granted access to other persons than the principal investigator and the co-investors are revoked. REDCap is maintained by the University Hospital Basel and has been approved for many other trials. Backups are automated every two weeks (if possible only incremental backup to limit data file size) to independent server. Backups are encrypted, independent of patients coding before. The key file is guarded by the principal investigator on his personal Clarunis drive. If necessary and useful the backup plans will be adjusted.

### Analysis and archiving

The REDCap project will be locked after eCRF data entry is completed, all data has been monitored and raised queries have been resolved. The complete study data set is exported from the REDCap database and transferred to the principal investigator and if applicable to the study statistician through a secure channel. The statistical analysis will be performed by the involved statistician at the University Hospital of Basel. The exported data will be archived for 10 years on a Clarunis server.

### Electronic and central data validation

Data entered into the REDCap platform will be validated for completeness and discrepancies automatically. In addition, the data will be reviewed by the responsible investigators. A regular download of the database will be performed and checked by the principal investigator and if applicable by the study statistician for missing values and correctness. All investigators have to respond to any resulting query and confirm or correct the corresponding data. Thereafter, the query can be closed.

## Monitoring

The sponsor will organize data monitoring by an independent revisor (hospital internal revisor). The revisor will visit the investigator’s site prior to the start of the study and during the study after inclusion the 5th participant and after inclusion of all patients. The sponsor will organize the monitoring visit. The revisor will have access to the source data and documents that are necessary to complete the CRF form. The revisor will verify the correct handling of the informed consent, the CRF and the conduction of the study according to the protocol and to GCP. He will control data handling as well. The DMC will give a short-written report to the sponsor for each visit.

## Audits and Inspections

No regular audit or inspection is planned for this trial. However, study documentation and the source data/documents are accessible to auditors/inspectors (also CEC and CA) and questions are answered during inspections. All involved parties must keep the participant data strictly confidential.

## Confidentiality, Data Protection

All data of this study are collected anonymously. The CRF forms are coded. Only investigators are able to know the identity of included patients. Direct access to source documents will be permitted for purpose of monitoring (12.3), audits and inspections (12.4) to the respective authorities.

## Storage of biological material and related health data

Not applicable for this trial.

# PUBLICATION AND DISSEMINATION POLICY

The results of this study will be published in a surgical peer-reviewed journal. Moreover, the results might be presented orally as a congress presentation. There are no restrictions to publication.

# FUNDING AND SUPPORT

## Funding

This study is funded by the sponsor. Due to the study participation no extra costs will be charged to the patients or their insurance companies. There will be no remuneration for study participants.

Consultations and surgery will not be performed for free. The indication for surgery is determined before the inclusion in this trial and therefore independent. The current standard is TEP. We calculated 400 € of additional costs for robotic instruments in rTAPP in comparison of TEP. These instruments will not be charged to the patients or their insurance companies. The excess costs of single use surgical material in the rTAPP group are reimbursed by Intuitive (Intuitive European Research Board, Intuitive Surgical, Chemin des Mûriers 1, 1170 Aubonne, Switzerland). Intuitive has no role in the study design, study accomplishment, analysis or interpretation. The maximum amount of instruments provided is determined to 40'000 €.

Patients will receive a stamped envelope in order to send the postoperative study questionnaires be letter mail.

We received a competitive grant from Intuitive (Intuitive European Research Board, Intuitive Surgical, Chemin des Mûriers 1, 1170 Aubonne, Switzerland) over 20'000 € to compensate for a study nurse and admin costs (ethics, publication).

## Other Support

Not applicable.

# INSURANCE

As a category A (KlinV Art. 61) study, no insurance is required. In the event of study-related damage or injuries, the liability of the institution Clarunis AG provides compensation, except for claims that arise from misconduct or gross negligence.

# REFERENCES

Provide a list of the references cited in the protocol.

1. Suguita, F. Y. et al. Learning curve takes 65 repetitions of totally extraperitoneal laparoscopy on inguinal hernias for reduction of operating time and complications. Surg. Endosc. 31, 3939–3945 (2017).

2. Lundström, K. J., Holmberg, H., Montgomery, A. & Nordin, P. Patient-reported rates of chronic pain and recurrence after groin hernia repair. Br. J. Surg. 105, 106–112 (2018).

3. Köckerling, F. & Schug-Pass, C. Early Surgical Intervention following Inguinal Hernia Repair with Severe Postoperative Pain. Front. Surg. 4, (2017).

4. Manangi, M., Shivashankar, S. & Vijayakumar, A. Chronic Pain after Inguinal Hernia Repair. Int. Sch. Res. Not. (2014) doi:10.1155/2014/839681.

5. Reinpold, W. Risk factors of chronic pain after inguinal hernia repair: A systematic review. Innovative Surgical Sciences (2020) doi:10.1515/iss-2017-0017.

6. Tolver, M. A., Rosenberg, J. & Bisgaard, T. Early pain after laparoscopic inguinal hernia repair. A qualitative systematic review. Acta Anaesthesiologica Scandinavica (2012) doi:10.1111/j.1399-6576.2011.02633.x.

7. Kingsnorth, A. & LeBlanc, K. Hernias: Inguinal and incisional. Lancet 362, 1561–1571 (2003).

8. Wu, J. J., Way, J. A., Eslick, G. D. & Cox, M. R. Transabdominal Pre-Peritoneal Versus Open Repair for Primary Unilateral Inguinal Hernia: A Meta-analysis. World J. Surg. 42, 1304–1311 (2018).

9. Aiolfi, A. et al. Primary inguinal hernia: systematic review and Bayesian network meta-analysis comparing open, laparoscopic transabdominal preperitoneal, totally extraperitoneal, and robotic preperitoneal repair. Hernia vol. 23 473–484 (2019).

10. Waite, K. E., Herman, M. A. & Doyle, P. J. Comparison of robotic versus laparoscopic transabdominal preperitoneal (TAPP) inguinal hernia repair. J. Robot. Surg. 10, 239–244 (2016).

11. Iraniha, A. & Peloquin, J. Long-term quality of life and outcomes following robotic assisted TAPP inguinal hernia repair. J. Robot. Surg. 12, 261–269 (2018).

12. Lyu, Y. et al. Comparison of endoscopic surgery and lichtenstein repair for treatment of inguinal hernias: A network meta-analysis. Medicine (United States) vol. 99 e19134 (2020).

13. Lydeking, L., Johansen, N., Oehlenschläger, J., Bay-Nielsen, M. & Bisgaard, T. Re-recurrence and pain 12 years after laparoscopic transabdominal preperitoneal (TAPP) or Lichtenstein’s repair for a recurrent inguinal hernia: a multi-centre single-blinded randomised clinical trial. Hernia 1–6 (2020) doi:10.1007/s10029-020-02139-0.

14. Köckerling, F. et al. What is the outcome of re-recurrent vs recurrent inguinal hernia repairs? An analysis of 16,206 patients from the Herniamed Registry. Hernia 1–9 (2020) doi:10.1007/s10029-020-02138-1.

15. Colvin, H. S., Rao, A., Cavali, M., Campanelli, G. & Amin, A. I. Glue versus suture fixation of mesh during open repair of inguinal hernias: A systematic review and meta-analysis. World J. Surg. 37, 2282–2292 (2013).

16. Rausa, E. et al. Open Inguinal Hernia Repair: A Network Meta-analysis Comparing Self-Gripping Mesh, Suture Fixation, and Glue Fixation. World Journal of Surgery vol. 43 447–456 (2019).

17. Scheuermann, U., Niebisch, S., Lyros, O., Jansen-Winkeln, B. & Gockel, I. Transabdominal Preperitoneal (TAPP) versus Lichtenstein operation for primary inguinal hernia repair - A systematic review and meta-analysis of randomized controlled trials. BMC Surg. 17, 55 (2017).

18. Hsu, W. et al. Preservation versus division of ilioinguinal nerve on open mesh repair of inguinal hernia: A meta-analysis of randomized controlled trials. World J. Surg. 36, 2311–2319 (2012).

19. Primatesta, P. & Goldacre, M. J. Inguinal hernia repair: Incidence of elective and emergency surgery, readmission and mortality. Int. J. Epidemiol. 25, 835–839 (1996).

20. Reinpold, W. et al. Retroperitoneal anatomy of the iliohypogastric, ilioinguinal, genitofemoral, and lateral femoral cutaneous nerve: consequences for prevention and treatment of chronic inguinodynia. Hernia (2015) doi:10.1007/s10029-015-1396-z.

21. Amid, P. K. Causes, prevention, and surgical treatment of postherniorrhaphy neuropathic inguinodynia: Triple neurectomy with proximal end implantation. Hernia (2004) doi:10.1007/s10029-004-0247-0.

22. Harms, B. A., Dehaas, D. R. & Starling, J. R. Diagnosis and Management of Genitofemoral Neuralgia. Arch. Surg. (1984) doi:10.1001/archsurg.1984.01390150071017.

23. Aasvang, E. & Kehlet, H. Chronic postoperative pain: The case of inguinal herniorrhaphy. British Journal of Anaesthesia (2005) doi:10.1093/bja/aei019.

24. Tolver, M. A., Strandfelt, P., Rosenberg, J. & Bisgaard, T. Pain characteristics after laparoscopic inguinal hernia repair. Surg. Endosc. (2011) doi:10.1007/s00464-011-1810-2.

25. Spivak, H. et al. Laparoscopic extraperitoneal inguinal hernia repair with spinal anesthesia and nitrous oxide insufflation. Surg. Endosc. (1999) doi:10.1007/s004649901161.

26. Köckerling, F. et al. TEP versus TAPP: comparison of the perioperative outcome in 17,587 patients with a primary unilateral inguinal hernia. Surg. Endosc. 29, 3750–3760 (2015).

27. Poelman, M. M. et al. EAES Consensus Development Conference on endoscopic repair of groin hernias. Surgical Endoscopy vol. 27 3505–3519 (2013).

28. Podolsky, D. & Novitsky, Y. Robotic Inguinal Hernia Repair. Surgical Clinics of North America vol. 100 409–415 (2020).

29. Techapongsatorn, S. et al. Mesh fixation technique in totally extraperitoneal inguinal hernia repair – A network meta-analysis. Surgeon 17, 215–224 (2019).

30. Mughal, A. et al. Laparoscopic-assisted transversus abdominis plane block as an effective analgesic in total extraperitoneal inguinal hernia repair: a double-blind, randomized controlled trial. Hernia 22, 821–826 (2018).

31. Yu, C. C. et al. A comprehensive study comparing tack and glue mesh fixation in laparoscopic total extraperitoneal repair for adult groin hernias. Surg. Endosc. 1–8 (2019) doi:10.1007/s00464-019-07234-7.

32. Olsen, M. F. et al. Pain relief that matters to patients: Systematic review of empirical studies assessing the minimum clinically important difference in acute pain. BMC Med. (2017) doi:10.1186/s12916-016-0775-3.

33. Olsson, A. et al. The Short-Form Inguinal Pain Questionnaire (sf-IPQ): An Instrument for Rating Groin Pain After Inguinal Hernia Surgery in Daily Clinical Practice. World J. Surg. 43, 806–811 (2019).

34. Charro, F. de & Rabin, R. EQ-SD: a measure of health status from the EuroQol Group. Ann. Med. (2001).

35. Brazier, J., Roberts, J. & Deverill, M. The Estimation of a Preference-Based Measure of Health from the SF-36 Address for correspondence : J. Health Econ. (2002).

36. Grewal, I. et al. Developing attributes for a generic quality of life measure for older people: Preferences or capabilities? Soc. Sci. Med. (2006) doi:10.1016/j.socscimed.2005.08.023.

37. Dindo, D., Demartines, N. & Clavien, P. A. Classification of surgical complications: A new proposal with evaluation in a cohort of 6336 patients and results of a survey. Annals of Surgery vol. 240 205–213 (2004).

38. Slankamenac, K., Graf, R., Barkun, J., Puhan, M. A. & Clavien, P. A. The comprehensive complication index: A novel continuous scale to measure surgical morbidity. Ann. Surg. 258, 1–7 (2013).

39. Monica, 1776 Main Street Santa & 90401-3208, C. 36-Item Short Form Survey (SF-36) | RAND. http://www.rand.org/health/surveys_tools/mos/36-item-short-form.html (2016).

40. Heniford, B. T. et al. Carolinas Comfort Scale as a Measure of Hernia Repair Quality of Life. Ann. Surg. 267, 171–176 (2018).

41. Hart, S. G. & Staveland, L. E. Development of NASA-TLX (Task Load Index): Results of Empirical and Theoretical Research. Adv. Psychol. 52, 139–183 (1988).

42. Hildebrandt, V. H., Bongers, P. M., Van Dijk, F. J. H., Kemper, H. C. G. & Dul, J. Dutch Musculoskeletal Questionnaire: Description and basic qualities. Ergonomics 44, 1038–1055 (2001).

43. Schulz, K. F., Altman, D. G. & Moher, D. CONSORT 2010 statement: Updated guidelines for reporting parallel group randomised trials. Int. J. Surg. 9, 672–677 (2011).

44. Aslam, S. & Emmanuel, P. Formulating a researchable question: A critical step for facilitating good clinical research. Indian Journal of Sexually Transmitted Diseases (2010) doi:10.4103/0253-7184.69003.

# APPENDICES

1. Case Report Form (CRF)
2. Patient Information and informed consent

**Supplementary Figures and Tables**

| **Item** | **TEP** | **rTAPP** |
| --- | --- | --- |
| Dilatation balloon plus suitable trocar | 445 | - |
| Trocars | 70 | 80 |
| Arm / Camera covers | 5 | 337 |
| Instruments | 231 | 931 |
| Glue for mesh fixation | 429 | - |
| Sutures | 5 | 34 |
| Verres needle |  | 8 |
| Mean OR time costs (unilateral repair, SD) | 1547 ± 458 | 1938 ± 497 |
| **Mean total costs (unilateral repair, SD)** | **2732 ± 458** | **3327 ± 497** |

**Table S1:** Overview of procedural costs in US dollars. TEP: Totally Extraperitoneal Plasty. rTAPP: robotic Transabdominal Preperitoneal Repair.

| **TABLE S2: EQ-5D-5L** | **TEP** N = 91*^1^* | **rTAPP** N = 91*^1^* | **p-value***^2^* |
| --- | --- | --- | --- |
| Screening | 0.82 (0.14) | 0.78 (0.15) | 0.069 |
| 24 Hours | 0.64 (0.18) | 0.64 (0.19) | 0.960 |
| 7 Days | 0.79 (0.11) | 0.79 (0.14) | 0.930 |
| 30 Days | 0.90 (0.10) | 0.90 (0.10) | 0.849 |
| 6 Months | 0.96 (0.09) | 0.94 (0.09) | 0.235 |
| 12 Months | 0.96 (0.09) | 0.95 (0.08) | 0.617 |
| *^1^*Mean (SD) | | | |
| *^2^*Welch Two Sample t-test | | | |

**Table S2:** European Quality of Life 5 Dimensions 5 Level Version (EQ-5D-5L) questionnaire results. TEP: Totally Extraperitoneal Plasty. rTAPP: robotic Transabdominal Preperitoneal Repair.

**References**

**-**
